# Integrated analyses of the transcriptome and small RNA of the hemiparasitic plant *Monochasma savatieri* before and after establishment of parasite-host association

**DOI:** 10.1186/s12870-021-02861-6

**Published:** 2021-02-10

**Authors:** Lanlan Chen, Qiaosheng Guo, Zaibiao Zhu, Hefang Wan, Yuhao Qin, Hui Zhang

**Affiliations:** Institute of Chinese Medicinal Materials, Nanjing Agricultural University, Nanjing, 210095 China

**Keywords:** High-throughput sequencing, Parasite-host association, Plant endogenous hormone, Root hemiparasite

## Abstract

**Background:**

*Monochasma savatieri* is a medicinal root hemiparasitic herb that extracts water and nutrients from the host plant via a haustorium. *M. savatieri* exhibits an enhanced growth after the establishment of parasite-host associations, but little is known about the molecular mechanism responsible. In this study, endogenous hormones, RNA sequencing and small RNA sequencing analysis were performed on *M. savatieri* before and after establishment of parasite-host associations.

**Results:**

When grown with the host, decreased contents of jasmonic acid (JA) and indole-3-acetic acid (IAA) and increased abscisic acid (ABA) content were observed in *M. savatieri* with the established parasitic relationship. When grown with the host, 46,424 differentially expressed genes (DEGs) and 162 differentially expressed miRNAs (DEmiRs) were identified in the comparison between *M. savatieri* with the established parasitic relationship and without the established parasitic relationship. Analysis of Gene Ontology (GO) and Kyoto Encyclopedia of Genes and Genomes (KEGG) showed that these DEGs and targets of DEmiRs mostly participated in plant hormone signal transduction, starch and sucrose metabolism, carbohydrate metabolism, cell growth and death, and transport and catabolism. Furthermore, correlation analysis of mRNA and miRNA revealed that 10 miRNA-target pairs from novel_mir65, novel_mir40, novel_mir80, miR397-5p_1, novel_mir36, novel_mir25 and novel_mir17 may have important roles in regulating the parasitic development of *M. savatieri*.

**Conclusions:**

Our study not only expands the understanding of enhanced growth in *M. savatieri* after the establishment of parasite-host associations, but also first provides abundant resources for future molecular and genetic studies in *M. savatieri*.

**Supplementary Information:**

The online version contains supplementary material available at 10.1186/s12870-021-02861-6.

## Background

*Monochasma savatieri* Franch. ex Maxim of Orobanchaceae is a perennial root hemiparasitic herb, which grows well in sunny and hilly terrains of Southeast China and Kyushu (Amakusa Islands), Japan [1–3]. After drying, its whole plant is necessary for Yanning syrup (one proprietary Chinese medicine), which is commonly applied to cure urinary tract infections, upper respiratory tract infections, and tonsillitis [4]. Currently, wild *M. savatieri* resources are decreasing sharply, mainly due to the unsustainable use of the wild resources [3]. So far, using tissue culture technique to micropropagate *M. savatieri* has been reported in a few studies [5, 6], and the lack of studies of parasite-host associations limits the establishment of its successful artificial cultivation system. In previous investigations, we studied the growth and anatomical characteristics of *M. savatieri* seedlings before and after establishing parasitic relationships with the host [7]. However, the research on the molecular mechanisms and regulatory networks of *M. savatieri* plants before and after establishing parasitic relationships with the host has not been carried out. This lack of knowledge limits the study for improving the successful parasitization of *M. savatieri* by using reliable technologies. Therefore, exploring the molecular mechanisms is needed to promote further study of the establishment of parasite-host associations to meet the increasing market demand.

As a special organ of parasitic plants, the haustorium is considered as the essence of parasitism [8]. In addition to haustorium-inducing factors, plant hormones also play roles in haustorium formation. In the process of haustorium formation in *Cassytha filiformis* (Lauraceae), the contents of cytokinins indolepropionic acid (IPA) and zeatin riboside (ZR) were 20 times and 4 times higher than the contents in the control part, respectively, after the parasitic plant attached to the host stem [9]. A study on *Cuscuta japonica* showed that the synthesized cytokinins were closely related to the development of the haustorium [10]. In addition, a large amount of auxin accumulated in the haustorium forming area of *Cuscuta* and was excreted from epidermal secretory cells [11].

As an economical and feasible sequencing technology, transcriptome sequencing (RNA-seq) has been widely used to expand sequence databases, and understand differentially expressed genes (DEGs) at the transcriptional and genomic levels. In recent years, RNA-seq has shown great potential in understanding the characteristics of parasitic plants. The dramatic expression patterns of host-specific genes were revealed by analyzing the data generated from RNA-seq of tissues at the interface between parasitic *Triphysaria versicolor* and its host *Medicago truncatula* or *Zea mays* [12]. In addition, comparative transcriptome analysis of three parasitic plants in Orobanchaceae indicated the conservation of chlorophyll synthesis in hemiparasites [13]. Ranjan et al. [14] sequenced the transcriptome of the parasitic weed *Cuscuta pentagona* and found that parasite genes encoding transporters and enzymes were up-regulated during the parasitic process, whereas genes associated with phytohormones were differentially expressed. RNA-seq was also used in a study on haustorium development of the root hemiparasite *Santalum album*, which suggested that auxin signaling was essential for haustorial initiation [15]. Kim et al. [16] used transcriptome sequencing to characterize mRNA transfer between *C. pentagona* and *Arabidopsis* or tomato hosts in a bidirectional manner.

MicroRNA (miRNA) composing of 21–24 nucleotides is a small non-coding RNA that primarily plays a role in translational repression or destabilization [17] by completely or partially binding the target mRNA [18]. In plants, miRNAs represent a group of the best studied small RNAs (sRNAs), and their various roles in development, including leaf growth, flowering and root elongation, have been reported [19, 20]. Recently, miRNAs have attracted attention as cross-species regulators [21, 22]. For example, functional studies in vitro and in vivo demonstrated that the exogenous plant miR168a in rice could regulate the expression of the mammalian gene LDLRAP1 [21]. A study in the parasite *Cuscuta campestris*-*Arabidopsis thaliana* pair identified 27 miRNAs of 22 nt in length, which targeted several *A. thaliana* mRNAs, that were up-regulated in the interface by taking advantage of sRNA sequencing technology [22].

In the present study, we took advantage of RNA-seq and sRNA sequencing technologies to profile the mRNAs and miRNAs in *M. savatieri* roots before and after establishing parasitic relationships to understand the mechanisms by which parasitizing the host promoted *M. savatieri* development. Therefore, the regulatory network of the interactions between DEmiRs and DEGs was constructed by analyzing the DEGs and differentially expressed miRNAs (DEmiRs) obtained from pairwise comparisons. The results of this study provide useful resources to explore the development of hemiparasites and further extend our understanding of miRNA-mRNA networks in plants.

## Results

### Endogenous hormone content

**Fig. 1:**
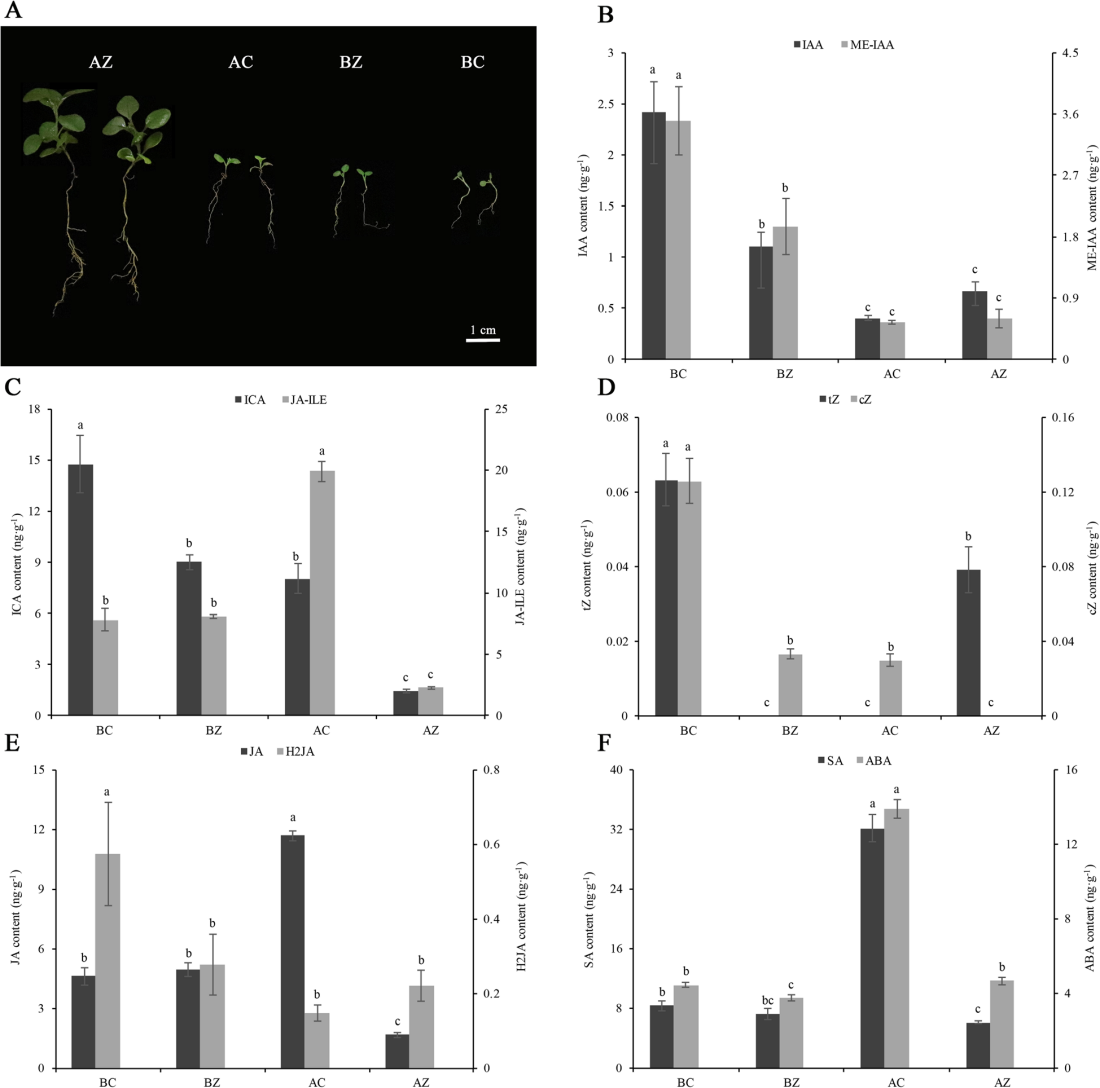
Endogenous hormone contents in *M. savatieri* grown with or without a host for 8 WAS and 16 WAS. **a** Morphological characteristics of *M. savatieri* seedlings in different groups. BC, growing without a host for 8 WAS; BZ, growing with one *G. jasminoides* for 8 WAS; AC, growing without a host for 16 WAS; AZ, growing with one *G. jasminoides* for 16 WAS. **b** Contents of IAA and ME-IAA. **c** Contents of ICA and JA-ILE. **d** Contents of tZ and cZ. **e** Contents of JA and H2JA. **f** Contents of SA and ABA. Different letters for the same indicator indicate statistically significant differences (*P* < 0.05). The error bars indicate the standard deviations of the three replicate determinations

To explore the mechanisms underlying the significant growth and development variations in *M. savatieri* before and after parasitizing *G. jasminoides* (Fig. 1a), we analyzed the endogenous hormone content of *M. savatieri* seedlings. When grown with or without a host, the content of indole-3-acetic acid (IAA), methyl indole-3-acetate (ME-IAA) and indole-3-carboxaldehyde (ICA) showed a consistently decreasing trend in the two developmental stages, and the content of ICA was higher than that of IAA and ME-IAA (Fig. 1b and c). At 8 WAS, the levels of trans-zeatin (tZ) and cis-zeatin (cZ) in *M. savatieri* seedlings grown without the host were the highest and were significantly different from the levels in any other samples (Fig. 1d). In the presence of the host, jasmonic acid (JA) content in *M. savatieri* parasitizing *G. jasminoides* showed a decrease of 66.20% compared with the JA content in the *M. savatieri* before establishing parasite-host associations. Additionally, there was a declining trend for dihydrojasmonic acid (H2JA) content after parasite-host associations were established, whereas no significant difference was found (Fig. 1e). At 8 WAS, there was no significant difference in salicylic acid (SA) content in *M. savatieri* seedlings in the presence or absence of *G. jasminoides*. At 16 WAS, the SA content of plants growing without *G. jasminoides* was significantly higher than the SA content of post-parasitization plants. In addition, the establishment of parasite-host associations significantly increased abscisic acid (ABA) content in plants compared with plants growing with *G. jasminoides* for 8 WAS (Fig. 1f).

### RNA-seq, sequence assembly and functional annotation

A total of twelve library reads of *M. savatieri* were built for RNA-seq to expose the transcriptome differences during parasite development using the BGISEQ-500 platform. In this study, 74.27 Gb of sequence data were obtained from roots of *M. savatieri* plants, which were grown without or with the host for 8 or 16 WAS. After assembly and de redundancy, 167,941 unigenes were obtained with a total length of 249,584,355 bp, a GC content of 40.76%, an N50 length of 2474 bp, and an average length of 1486 bp. The sequence length of these unigenes was between 200 bp and 3000 bp, and up to 86,644 unigenes (51.59%) were larger than 1000 bp (Fig. S1). The ratio of clean reads to total raw reads in all cDNA libraries exceeded 95.85%, and the quality of the sequenced bases is presented in Table 1. The lowest value of Q20 was 97.62%, and the lowest value of Q30 was 89.72%. The quality of assembled transcripts was evaluated by BUSCO, and the results are shown in Fig. S2. The BUSCO analysis showed that among all the assembled unigenes, of which 22% were single-copy complete, 76% were duplicated complete, 1% were fragmented, and 1% were missing.

**Table 1:** Statistical summary of the transcriptome assembly for *M. savatieri*

Sample	Total raw reads (M)	Total clean reads (M)	Clean reads number	Clean reads ratio(%)	Q20(%)	Q30(%)	Total mapping(%)
BC 1	64.44	61.88	61,877,098	96.03	97.71	90.11	89.39
BC 2	64.44	61.94	61,944,568	96.13	97.73	90.28	89.36
BC 3	64.4	61.84	61,843,080	96.03	97.83	90.53	84.9
BZ 1	64.44	61.73	61,727,048	95.79	97.71	90.17	89.88
BZ 2	64.44	61.93	61,932,718	96.11	97.81	90.51	89.7
BZ 3	64.43	62.01	62,009,278	96.24	97.83	90.54	87.89
AC 1	64.4	62.03	62,029,070	96.31	97.83	90.47	89.16
AC 2	64.44	61.94	61,937,642	96.12	97.78	90.31	88.4
AC 3	64.41	61.74	61,742,462	95.85	97.62	89.72	90.01
AZ 1	64.44	61.79	61,785,376	95.88	97.63	89.85	89.67
AZ 2	64.4	61.86	61,859,516	96.06	97.78	90.34	90.7
AZ 3	64.46	62.16	62,155,874	96.43	97.88	90.7	87.79

BC, growing without a host for 8 WAS; BZ, growing with one *G. jasminoides* for 8 WAS; AC, growing without a host for 16 WAS; AZ, growing with one *G. jasminoides* for 16 WAS

To obtain an overall understanding of the functions of the unigenes that were obtained, all unigenes were searched against the Clusters of Orthologous Groups for Eukaryotic Complete Genomes (KOG), SwissProt, non-redundant protein database (NR), KEGG, nucleotide sequence database (NT) and the Pfam databases. Approximately 116,572 (blast to NR database), 95,387 (blast to NT database), 94,681 (blast to KEGG database), and 91,301 (blast to Pfam database) unigenes were similar in sequence to known genes (Table 2). By GO analysis, 62,227 unigenes were annotated and classified into 41 functional groups of three GO categories (Fig. S2). Among the GO terms, catalytic activity and molecular function accounted for the largest proportion in functional groups and GO categories, respectively.

**Table 2:** Statistical summary of functional annotation of *M. savatieri* unigenes

Annotated databases	Annotated number	Annotated percentage
NR	116,572	69.41%
NT	95,387	56.80%
Swissprot	88,877	52.92%
KEGG	94,681	56.38%
KOG	96,800	57.64%
Pfam	91,301	54.36%
GO	62,227	37.05%
Total Annotated	126,048	75.05%

### Identification of DEGs

**Fig. 2:**
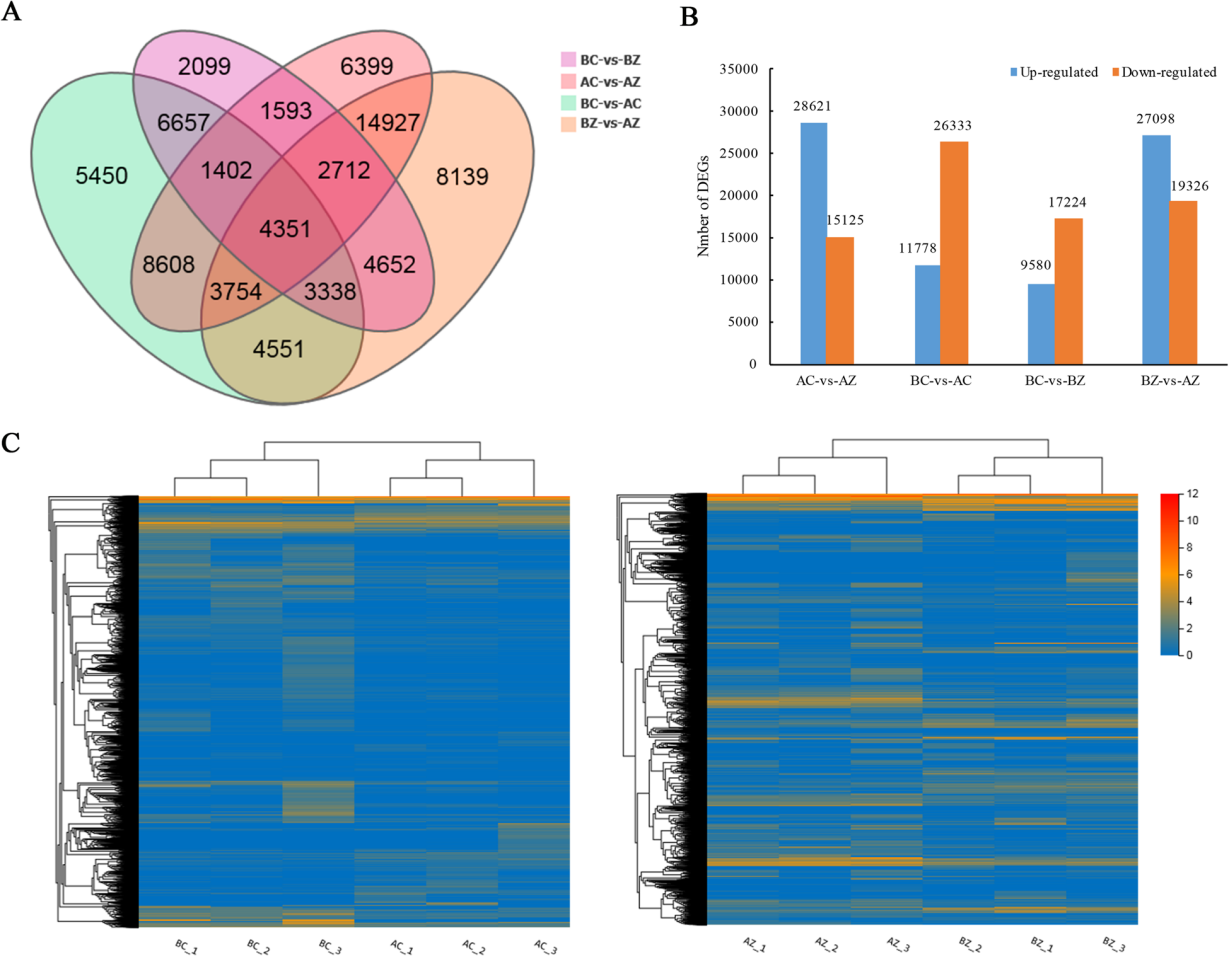
Gene expression profiles of *M. savatieri* grown with or without a host for 8 WAS and 16 WAS. **a** Venn diagram of common and unique DEGs among different comparisons. **b** Expression patterns of DEGs among different comparisons. **c** Expression profiles and cluster analysis of total DEGs in BC/AC and BZ/AZ comparisons. BC, growing without a host for 8 WAS; BZ, growing with one *G. jasminoides* for 8 WAS; AC, growing without a host for 16 WAS; AZ, growing with one *G. jasminoides* for 16 WAS

To present the number of DEGs in different comparisons, a Venn diagram was built (Fig. 2a). Among these four comparisons, BZ/AZ had the largest number of DEGs (46,424), followed by AC/AZ (43,746) and BC/AC (38,111), and BC/BZ had the smallest number of DEGs (26,804). In addition, 4351 DEGs were identified in all four comparisons. There were 11,778 up-regulated genes and 26,333 down-regulated genes detected under AC compared with BC. Comparison of BZ with AZ revealed 27,098 up-regulated genes and 19,326 down-regulated genes (Fig. 2b and Additional file 2). To have a visual understanding of the overall expression profile, a heat map was constructed (Fig. 2c). By analyzing gene expression, a large portion of genes with higher levels in BC were found to exhibit lower levels in AC. Furthermore, most of the DEGs presented larger expression differences in BZ and AZ than in BC and AC.

### Functional classification and enrichment analysis of the DEGs

**Fig. 3:**
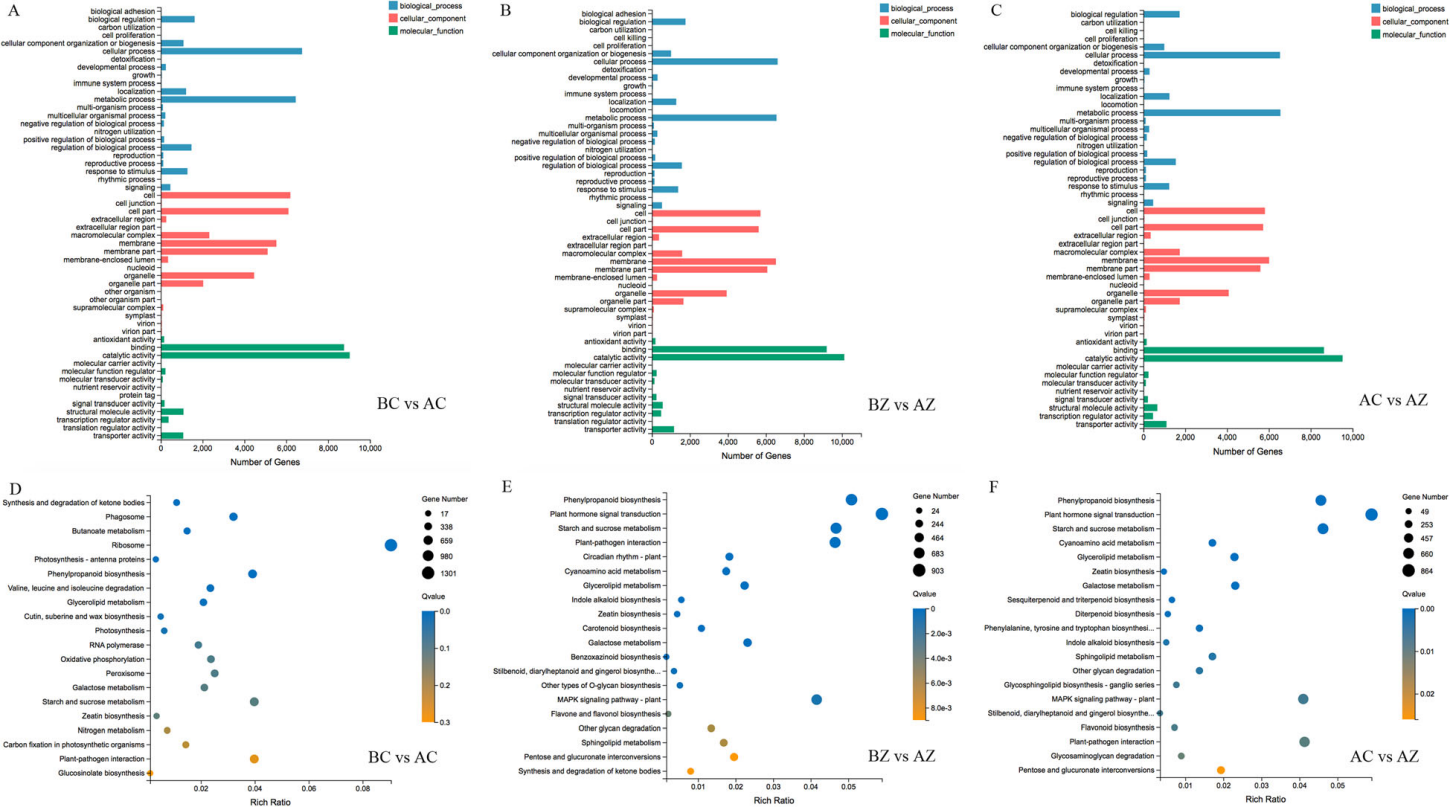
GO analysis and KEGG pathway enrichment analysis of DEGs in *M. savatieri*. The bar graphs show the GO analysis of DEGs in BC/AC (**a**), BZ/AZ (**b**) and AC/AZ (**c**) comparisons. The bubble charts show the KEGG pathway enrichment analysis of DEGs in BC/AC (**d**), BZ/AZ (**e**) and AC/AZ (**f**) comparisons. In the bubble charts, the X-axis represents the rich factor. The Y-axis represents the KEGG pathway. The color of the dots indicates the Qvalue. The size of the dots indicates the number of enriched DEGs of the pathway. BC, growing without a host for 8 WAS; BZ, growing with one *G. jasminoides* for 8 WAS; AC, growing without a host for 16 WAS; AZ, growing with one *G. jasminoides* for 16 WAS

To further understand the DEGs, we used GO and KEGG pathway analysis to evaluate the DEGs (Fig. 3). By analyzing these three comparisons, the annotated DEGs were observed to be enriched for several specific GO terms. The number of DEGs in the BZ vs AZ comparison was greater than the number of DEGs in the BC vs AC and AC vs AZ comparisons. With regard to each comparison, most of the DEGs were enriched in the cellular component category, with the highly represented GO terms classified into “cell”, “membrane”, “cell part”, “organelle” and “membrane part”. The GO terms were significantly classified into “metabolic process” and “cellular process” in the biological process category and “catalytic activity” and “binding” in the molecular function category (Fig. 3a-c and Additional file 3).

To gain more insights into the metabolic pathways and biological functions of the DEGs, an enrichment analysis was performed on the basis of the KEGG database. There were 14,375 DEGs detected in the BC vs AC comparison, 15,320 DEGs in the BZ vs AZ comparison, and 14,659 DEGs in the AC vs AZ comparison. In these comparisons, the top 20 pathways that were enriched by the most significant differentially expressed genes are given (Fig. 3d-f). In the BZ vs AZ and AC vs AZ comparisons, the DEGs were mostly enriched in “plant hormone signal transduction”, “starch and sucrose metabolism”, “phenylpropanoid biosynthesis”, “MAPK signaling pathway-plant”, “galactose metabolism”, “zeatin biosynthesis”, and “plant-pathogen interaction”. Notably, “circadian rhythm-plant” and “carotenoid biosynthesis” were only enriched in the BZ vs AZ comparison. In the BC vs AC comparison, the DEGs were significantly enriched in “photosynthesis-antenna proteins”, “phagosome”, “butanoate metabolism”, “ribosome”, and “synthesis and degradation of ketone bodies”. Moreover, “nitrogen metabolism”, “carbon fixation in photosynthetic organisms”, “photosynthesis”, and “cutin, suberine and wax biosynthesis” were significantly enriched only in the BC vs AC comparison.

### Transcription factors related to the biological processes before and after establishing parasitic relationship with the host

Since *M. savatieri* established a parasitic relationship with the host during growth with *G. jasminoides*, the DEGs related to the biological processes in the BZ vs AZ comparison were then selected for further analysis. Here, 66 transcription factors (TFs), which might have the putative regulatory role in the establishment of parasite-host associations, were identified among these DEGs. The TF families included WRKY, TCP, PBF-2-like, NAC, MYB, MADS, GRAS, G2-like, FAR1, C3H, C2H2, C2C2-GATA, C2C2-Dof, BES1, ARF, AP2-EREBP, and ABI3VP1 (Table S1). Among these TFs, the MYB family contained the largest number of DEGs, of which 7 putative MYBs were up-regulated under BZ/AZ. The WRKY family had 8 DEGs, which followed the MYB family. Moreover, the expression levels of all 8 putative WRKYs decreased under AZ compared to BZ, suggesting that the TFs may negatively regulate the establishment of parasite-host associations.

### DEG network and pathways related to establishment of the parasitic relationship

**Fig. 4:**
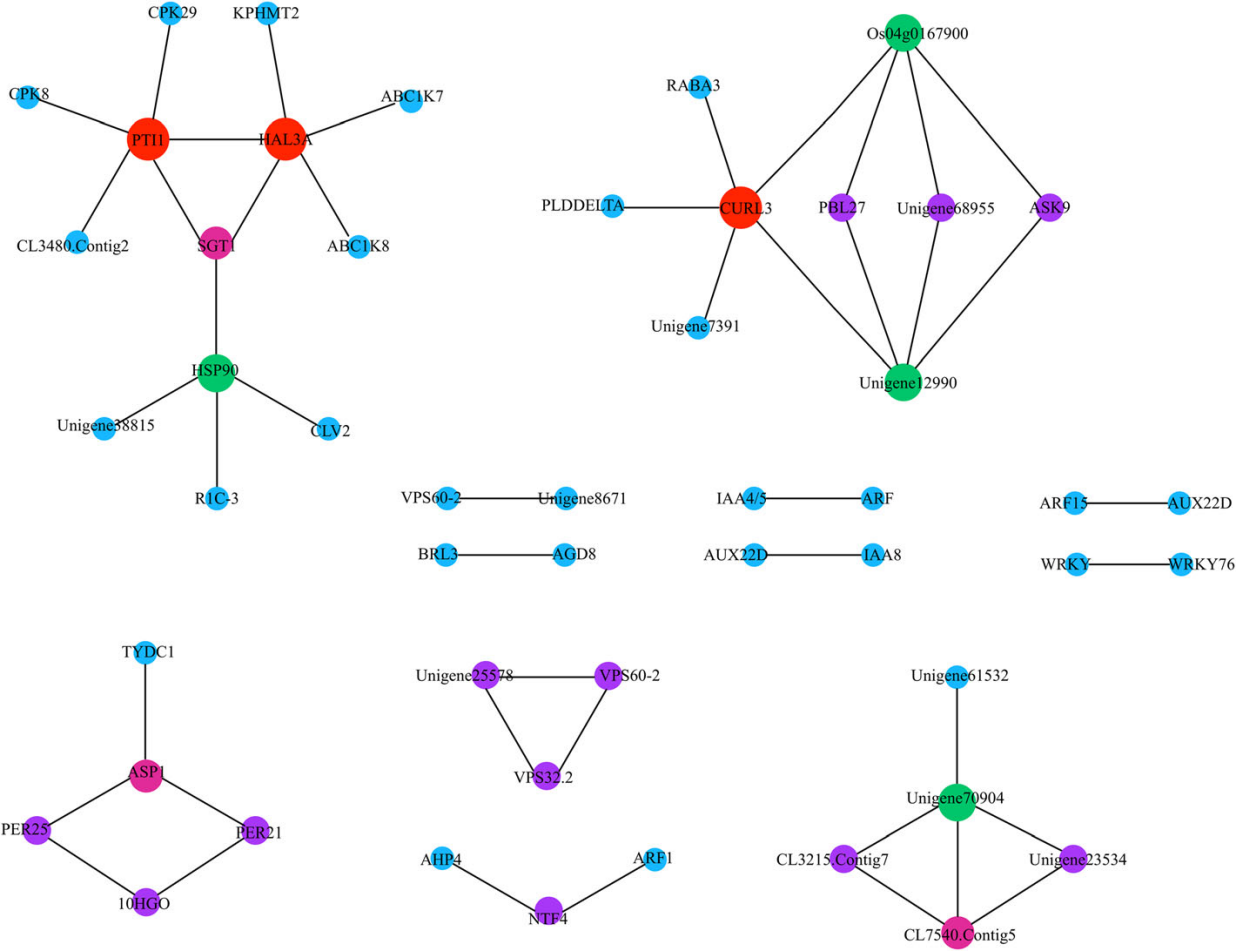
DEG networks associated with the establishment of parasite-host associations in *M. savatieri*. The size and color of the nodes indicate the interaction of the gene. Nodes are labeled annotations from the *M. savatieri* RNA-seq database

To understand the underlying mechanism of *M. savatieri-*enhanced parasitic development, the DEGs only specifically expressed in the BZ vs AZ comparison and significantly enriched in “plant-pathogen interaction”, “carotenoid biosynthesis”, “phenylpropanoid biosynthesis”, “plant hormone signal transduction”, and “endocytosis” were compared with the STRING database by DIAMOND, and the interaction between genes was obtained by homology with known proteins. In total, 46 interactions among 50 genes were identified from 500 DEGs in the rebuilt gene network (Fig. 4). Among the 50 identified genes, three DEGs, namely, *MsPTI1*, *MsHAL3A*, and *MsCURL3*, interacted with other DEGs more frequently, indicating that they may have key roles in the establishment of the *M. savatieri*-host association.

**Fig. 5:**
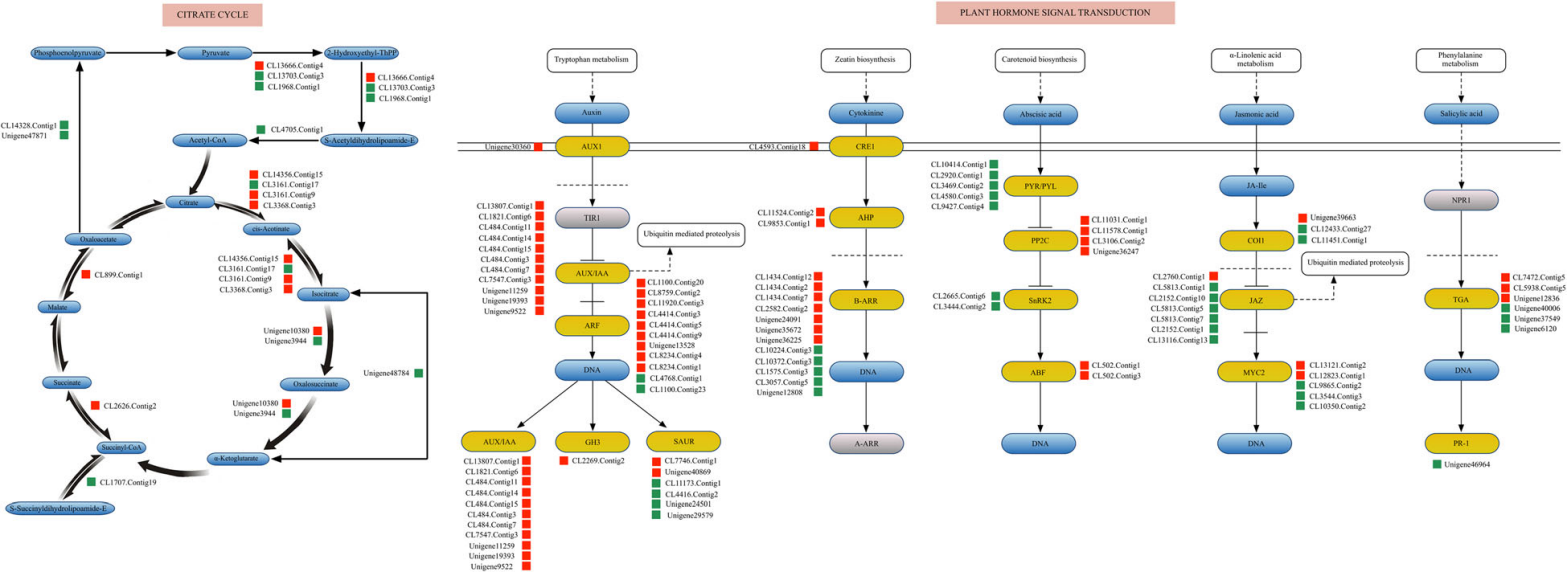
Transcriptional changes in genes related to the TCA cycle and plant hormone signal transduction in *M. savatieri* before and after establishment of parasite-host association. The blue rounded rectangle represents compounds, the yellow rounded rectangle represents enzymes, proteins or transcriptional factors, and the gray rounded rectangle represents not detected. The red squares represent up-regulated DEGs under AZ compared to BZ, whereas green squares represent down-regulated DEGs. BZ, growing with one *G. jasminoides* for 8 WAS; AZ, growing with one *G. jasminoides* for 16 WAS

By mapping to the KEGG database, the three DEGs *MsPTI1*, *MsHAL3A*, and *MsCURL3* were found to be involved in “metabolic pathways” and “plant hormone signal transduction”. *M. savatieri* exhibited enhanced growth after establishing parasitic relationships with the host, and the above results indicated that plant hormones played important roles in the establishment of *M. savatieri*-host associations. Thus, the DEGs only specifically expressed in the BZ vs AZ comparison were listed in the “plant hormone signal transduction” and “citrate cycle” pathways to obtain detailed information (Fig. 5). In the citrate cycle, 8 DEGs (4 up-regulated and 4 down-regulated) were involved in the processes of the cycle between citrate, cis-acotinate and isocitrate and biosynthesis of acetyl-CoA. In plant hormone signal transduction, the number of genes involved in auxin signal transduction was the highest, and the expression levels of these genes were up-regulated after establishing parasitic relationships with the host. In addition, 2 *MsCRE1* putative target genes (CL11524. Contig2 and CL9853. Contig1) were identified in cytokinine signal transduction.

### qRT-PCR validation of DEGs associated with parasite development

Establishing parasitic relationships with the host was achieved by a series of processes, and numerous genes were involved in these steps. In the experiment, we performed validation of selected 14 DEGs by quantitative real-time PCR (qRT-PCR). The selected DEGs were predicted to play a role in starch and sucrose metabolism (*Unigene11723*, *Unigene28565*, *Unigene25653*, *Unigene35991*, *Unigene32595*), hormone signal transduction (*Unigene9451*, *Unigene21852*, *Unigene29438*, *Unigene60184*), and cell part (*Unigene36901*, *Unigene470*, *Unigene5067*, *Unigene10023*, *Unigene25917*). The qRT-PCR analysis showed that the expression patterns of these genes were consistent with the expression pattern of the corresponding transcriptome data (Fig. S3).

Among the genes related to starch and sucrose metabolism (Fig. S3a and b), the expression levels of *Unigene11723* and *Unigene28565* increased under AZ compared with BZ and AC. The expression of *Unigene25653*, *Unigene35991* and *Unigene32595* decreased under AZ relative to BZ and AC. The expression levels of four genes involved in hormone signal transduction were significantly different before and after establishing parasitic relationships with the host (Fig. S3c and d). In addition, qRT-PCR analysis showed that *Unigene9451*, *Unigene21852* and *Unigene29438* exhibited much higher expression levels in AZ than the other three groups, while regarding *Unigene60184*, its expression level decreased at least 20-fold. In the cell part, four genes (*Unigene36901*, *Unigene470*, *Unigene5067* and *Unigene10023*) exhibited a higher expression level in AZ than AC, while *Unigene25917* exhibited a significantly lower expression level in AZ than BZ (Fig. S3e and f).

### Small RNA sequencing and category annotation

A total of twelve sRNA libraries (BC, BZ, AC, and AZ, each repeated three times) of *M. savatieri* were prepared from the roots of *M. savatieri* to reveal the miRNA differences in parasite development. In the present study, 21,527,722 to 30,095,656 raw tags were generated from each library, and 20,667,261 to 28,872,904 clean tags per library were obtained after removing low-quality tags (Table 3). The lowest value of Q20 was 98.6%, and the percentage of mapped tags in all sRNA libraries exceeded 41.1%. The sequence length of these small RNAs ranged from 15 nt to 30 nt, of which the number of small RNAs with a length of 24 nt was the largest (Fig. S4). According to the Rfam and GenBank databases, we annotated the sequences from all *M. savatieri* libraries and described the counts of different categories (Table 4). Among these libraries, sequences matching to mature sRNA were the most abundant class, which accounted for almost 2–11% after excluding the unmapping sequences.

**Table 3:** Statistical summary of small RNA sequencing data for *M. savatieri*

Sample	Raw tag count	Short valid length tag	Clean tag count	Q20 of clean tag (%)	Percentage of clean tag (%)	Mapped tag	Mapped tag percentage (%)
BC 1	28,103,827	1,137,232	26,100,927	98.6	92.87	14,883,410	57.02
BC 2	28,343,426	1,214,022	26,039,115	99.1	91.87	15,582,358	59.84
BC 3	29,717,549	372,975	26,924,615	99.1	90.6	12,956,213	48.12
BZ 1	30,095,656	471,619	28,587,339	99.1	94.99	16,375,175	57.28
BZ 2	28,294,198	1,028,007	26,246,425	99.2	92.76	17,828,594	67.93
BZ 3	21,527,722	71,884	20,667,261	99.0	96	10,207,888	49.39
AC 1	28,378,126	180,436	26,906,040	99.1	94.81	12,476,677	46.37
AC 2	28,251,907	119,769	27,298,942	99.1	96.63	12,509,082	45.82
AC 3	29,622,162	109,009	28,559,486	99.0	96.41	14,494,379	50.75
AZ 1	29,288,996	85,248	28,368,014	98.9	96.86	11,660,443	41.10
AZ 2	29,997,041	113,593	28,872,904	99.1	96.25	13,248,923	45.89
AZ 3	29,759,347	104,677	28,539,455	99.0	95.9	11,913,184	41.74

BC, growing without a host for 8 WAS; BZ, growing with one *G. jasminoides* for 8 WAS; AC, growing without a host for 16 WAS; AZ, growing with one *G. jasminoides* for 16 WAS

**Table 4:** Summary of general annotation of clean sequences for small RNAs in *M. savatieri*

Type		mature	snRNA	rRNA	snoRNA	tRNA	intron	intergenic	precursor	unmap
BC-1	Count	1,819,316	8499	154,007	2712	1459	187	89,351	17,487	11,181,851
BC-2	Count	1,885,269	5196	167,570	2252	1270	177	198,832	20,579	10,417,322
BC-3	Count	710,966	12,272	316,971	4057	5614	389	166,410	8084	13,915,066
BZ-1	Count	3,227,181	10,792	132,903	2024	1379	672	231,326	15,850	12,178,998
BZ-2	Count	1,529,809	6917	244,911	3695	1386	117	117,732	19,097	8,381,136
BZ-3	Count	511,765	10,647	363,318	6421	7966	152	165,638	7130	10,418,194
AC-1	Count	489,518	13,630	274,587	3992	9445	1321	208,337	6475	14,381,159
AC-2	Count	1,151,818	11,542	130,686	2078	3102	1049	288,352	9469	14,760,687
AC-3	Count	1,109,687	9683	205,523	2015	2559	297	216,271	21,169	14,026,941
AZ-1	Count	637,039	22,516	309,291	4168	12,149	243	194,864	9588	16,651,139
AZ-2	Count	823,625	19,333	351,638	3178	6251	128	292,921	13,832	15,578,629
AZ-3	Count	855,925	18,067	220,546	2907	4628	177	286,809	10,854	16,588,191

BC, growing without a host for 8 WAS; BZ, growing with one *G. jasminoides* for 8 WAS; AC, growing without a host for 16 WAS; AZ, growing with one *G. jasminoides* for 16 WAS

### Discovery of known and novel miRNAs

**Fig. 6:**
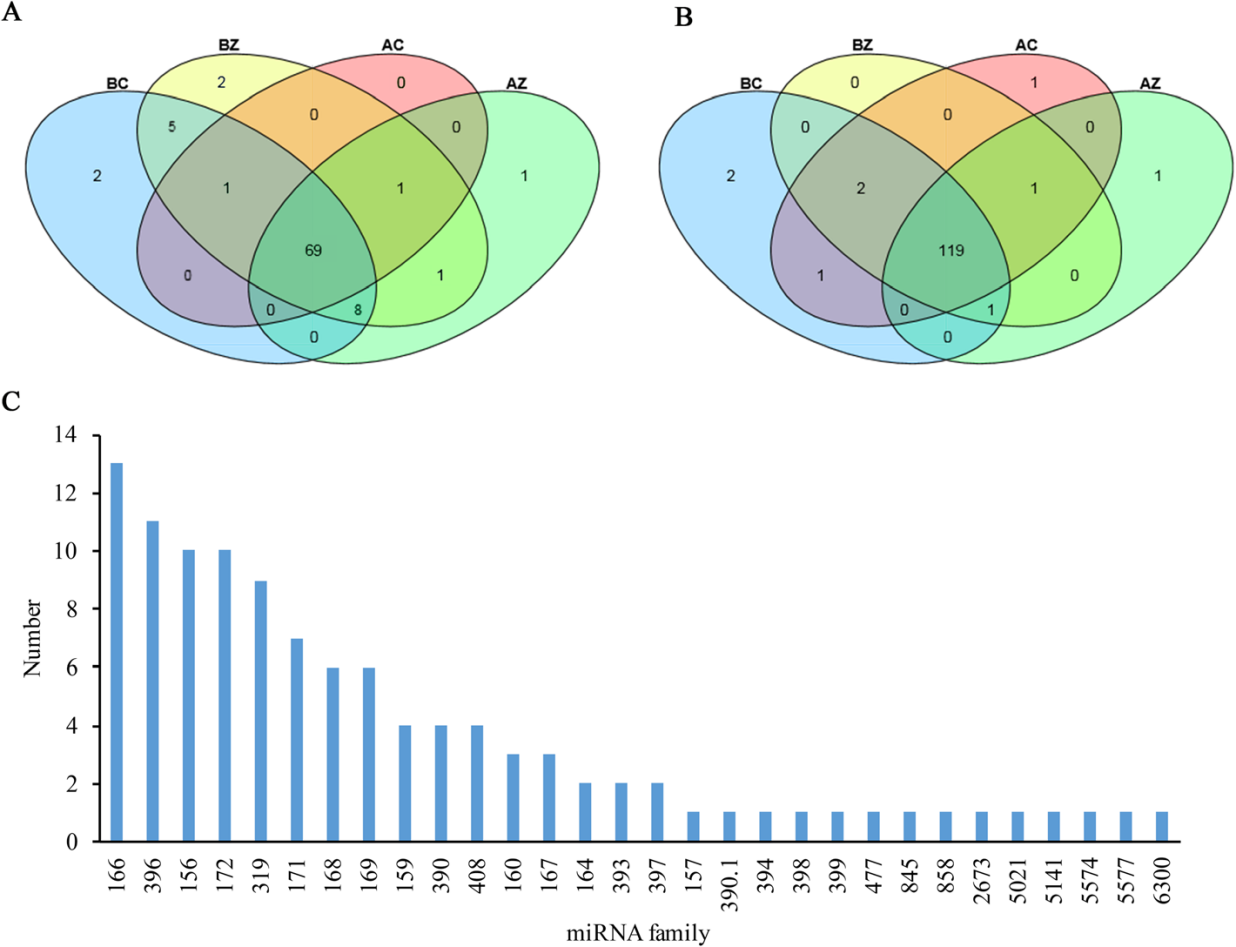
Identification of miRNAs in *M. savatieri*. **a** Venn diagrams of known miRNAs. **b** Venn diagrams of novel miRNAs. **c** Number of known miRNAs in each miRNA family. BC, growing without a host for 8 WAS; BZ, growing with one *G. jasminoides* for 8 WAS; AC, growing without a host for 16 WAS; AZ, growing with one *G. jasminoides* for 16 WAS

There were 110 known miRNAs identified from small RNA sequences of *M. savatieri* after comparing the sequences with the known mature miRNAs in the miRBase. With the use of miRA, 128 sequences were identified as candidates for novel miRNAs. As shown in Fig. S5, the first base distribution of known and novel miRNAs in *M. savatieri* was analyzed. In the identified miRNAs, the percent of the first base was not evenly distributed. The U percent in the first base of most known and novel miRNAs was more than 25%. Venn diagrams were constructed to represent the numbers of overlapping and specific miRNAs from each group (Fig. 6a and b). Among the four libraries, the number of known and novel miRNAs identified in each library was 69 and 119, respectively. Family analysis revealed that of the known miRNAs in *M. savatieri*, 110 miRNAs were from 30 families (Fig. 6c). Among them, the miR166 family had 13 miRNA members and was the largest family of miRNAs, followed by the miR396 family, which had 11 miRNA members. In addition, 14 families contained only one miRNA member, such as miR157 and miR398 (Fig. 6c).

### Screening for DEmiRs

**Fig. 7:**
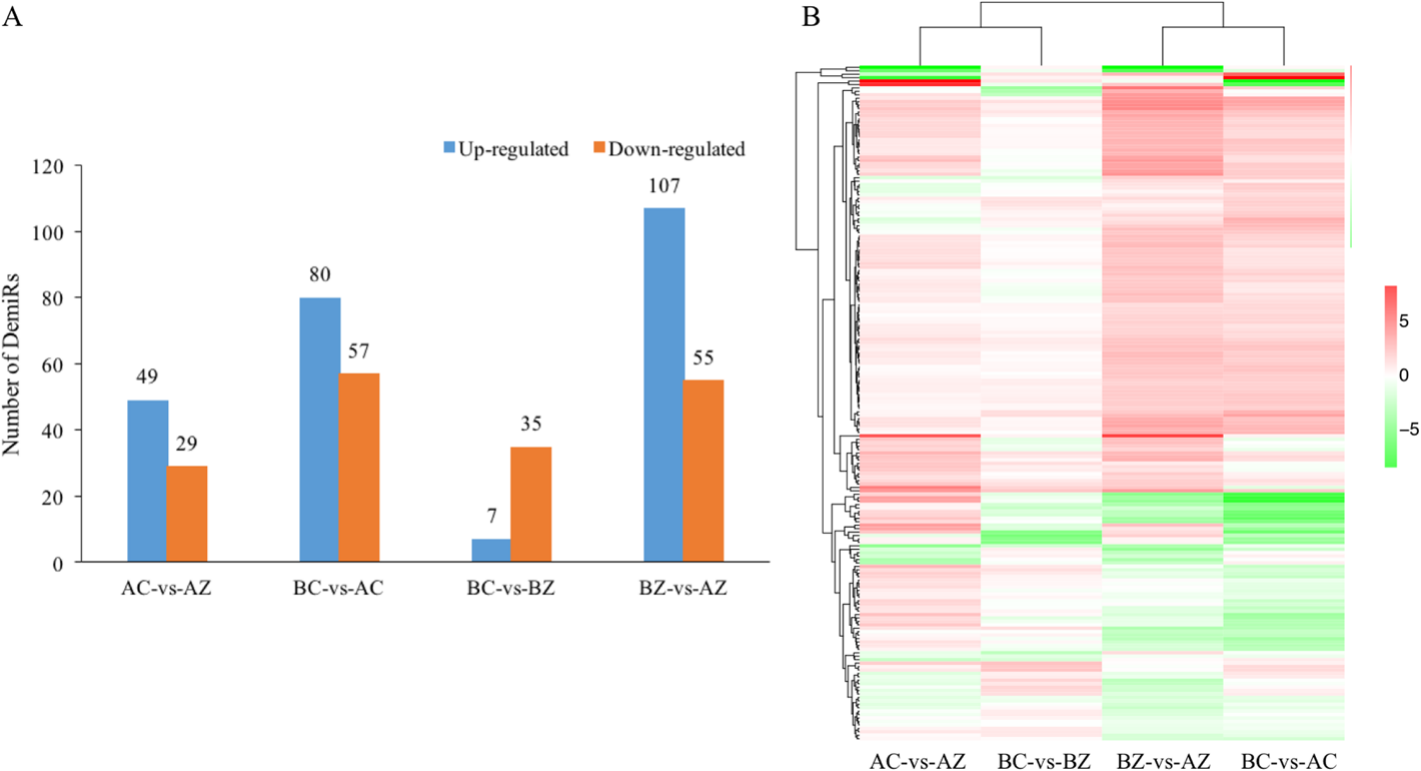
Changes in miRNA expression profiles of *M. savatieri* before and after establishment of parasite-host association. **a** The expression patterns of DEmiRs among different comparisons. **b** Cluster analysis of total DEmiRs among different comparisons. BC, growing without a host for 8 WAS; BZ, growing with one *G. jasminoides* for 8 WAS; AC, growing without a host for 16 WAS; AZ, growing with one *G. jasminoides* for 16 WAS

To determine the differences in miRNA expression in *M. savatieri* before and after establishing parasitic relationship with the host, comparative analyses were performed between AC and AZ, BC and AC, BC and BZ, and BZ and AZ. In general, 198 DEmiRs were detected by using DEGseq. In comparison with AC, the number of down-regulated and up-regulated miRNAs detected in the AZ was 29 and 49, respectively. Comparison of BZ with AZ revealed 55 down-regulated miRNAs and 107 up-regulated miRNAs in the AZ (Fig. 7a and Additional file 4). In addition, a general overview of the DEmiRs screened between the four comparison groups was visualized in a heat map (Fig. 7b). According to the expression analysis, most of the DEmiRs under the BC vs AC comparison were found to have similar expression patterns under the BZ vs AZ comparison. In addition, most DEmiRs exhibited larger expression differences in the AC vs AZ comparison than in the BC vs BZ comparison.

### Functional annotation of DEmiR target genes

**Fig. 8:**
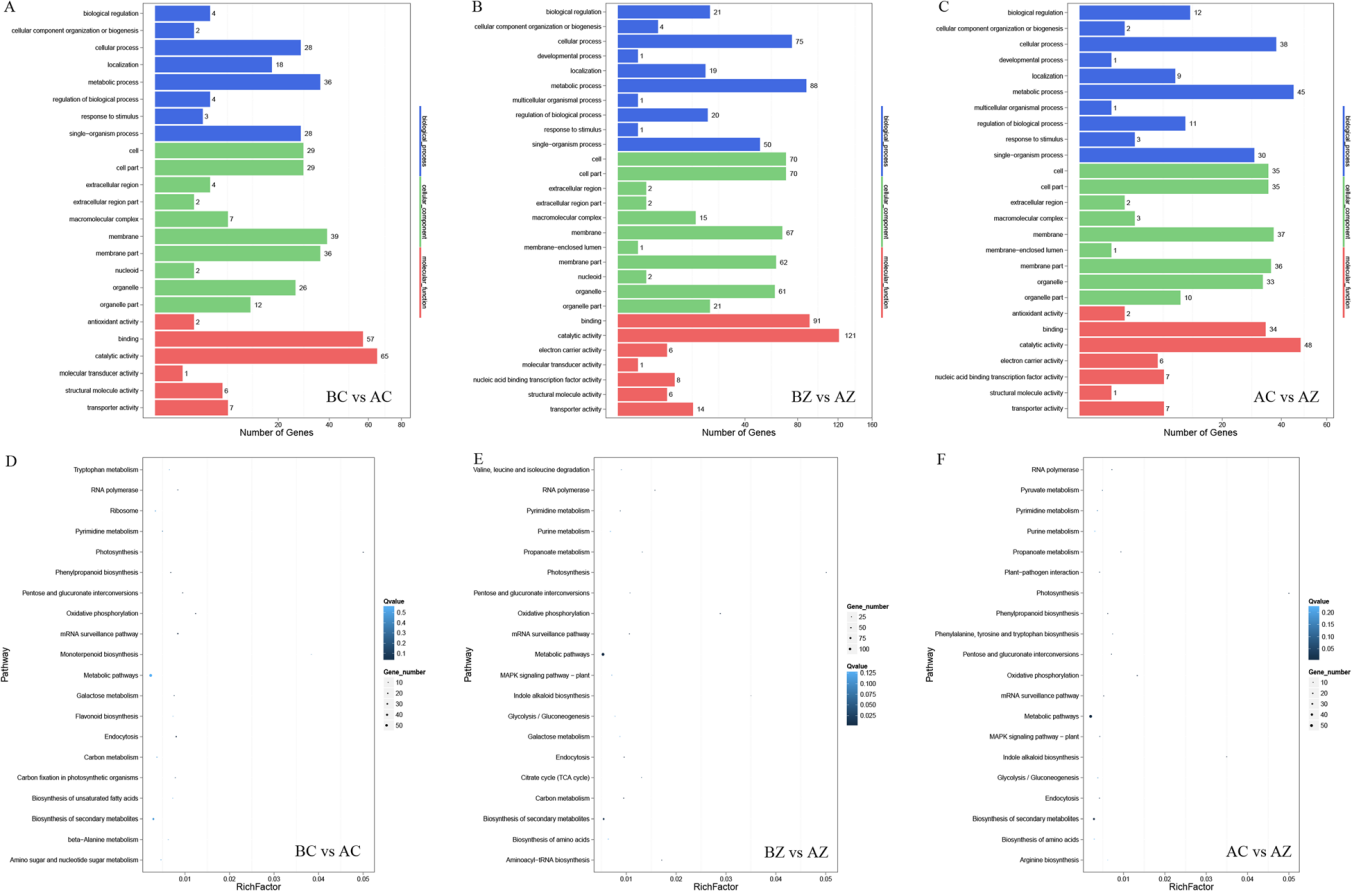
GO analysis and KEGG pathway enrichment analysis of DEmiR target genes in *M. savatieri*. The bar graphs show the GO analysis of DEmiR target genes in BC/AC (**a**), BZ/AZ (**b**) and AC/AZ (**c**) comparisons. The bubble charts show the KEGG pathway enrichment analysis of DEmiR target genes in BC/AC (**d**), BZ/AZ (**e**) and AC/AZ (**f**) comparisons. In the bar graphs, the number shown on the abscissa is obtained after taking the square root. In the bubble charts, the X-axis represents the rich factor. The Y-axis represents the KEGG pathway. The color of the dots indicates the Qvalue. The size of the dots indicates the number of enriched DEGs of the pathway. BC, growing without a host for 8 WAS; BZ, growing with one *G. jasminoides* for 8 WAS; AC, growing without a host for 16 WAS; AZ, growing with one *G. jasminoides* for 16 WAS

To further understand the DEmiRs, we used GO and KEGG pathway analysis to evaluate the DEmiR target genes (Fig. 8). By analyzing the three comparisons, the annotated DEmiR target genes were observed to be enriched for several specific GO terms. The number of annotated DEmiR targets in the BZ vs AZ comparison was greater than the number of annotated DEmiR targets in the BC vs AC and AC vs AZ comparisons. With regard to each comparison, most DEmiR targets were enriched in the cellular component category, with the highly represented GO terms classified into “cell”, “membrane”, “cell part”, “organelle” and “membrane part”. The GO terms were significantly classified into “single-organism process”, “metabolic process” and “cellular process” in the biological process category and “catalytic activity” and “binding” in the molecular function category (Fig. 8a-c).

In the KEGG pathway, the number of DEmiR targets identified in the BC vs AC, BZ vs AZ, and AC vs AZ comparisons were 115, 197, and 85, respectively (Additional file 5). The top 20 pathways that were enriched by the most significant DEmiR targets in the three comparisons are given (Fig. 8d-f). Among all these comparisons, the DEmiR targets were mostly enriched in “RNA polymerase”, “metabolic pathways”, “biosynthesis of secondary metabolites”, “endocytosis”, “oxidative phosphorylation”, “photosynthesis”, and “pentose and glucuronate interconversions”. Notably, “purine metabolism”, “propanoate metabolism”, “MAPK signaling pathway-plant”, “indole alkaloid biosynthesis”, “glycolysis/gluconeogenesis” and “biosynthesis of amino acids” were both enriched in the BZ vs AZ and AC vs AZ comparisons. Moreover, “ribosome”, “carbon fixation in photosynthetic organisms”, “monoterpenoid biosynthesis” and “flavonoid biosynthesis” were significantly enriched only in the BC vs AC comparison.

### qRT-PCR validation of DEmiRs and targets associated with parasite development

Through qRT-PCR, 6 known (miR172a_3, miR156_2, miR396g-3p, miR171b-3p_3, miR397-5p_1, miR396h) and 9 novel (novel_mir80, novel_mir50, novel_mir46, novel_mir113, novel_mir99, novel_mir49, novel_mir87, novel_mir40, novel_mir65) DEmiRs were selected to analyze the expression levels. These selected miRNA targets were predicted to be associated with biological regulation, membrane and organelle, and metabolism. Among biological regulation-related miRNAs (Fig. S6a and b), the expression levels of novel_mir50 and miR396g-3p increased substantially in *M. savatieri* seedlings after parasitizing *G. jasminoides*, while the expression level of novel_mir80 showed a large decrease. With regard to membrane- and organelle-related miRNAs (Fig. S6c and d), the expression of miR171b-3p_3 decreased under AZ relative to BZ and AC, while the expression of novel_mir99 showed a significant increase after establishment of parasite-host association. The five metabolism-related miRNAs (novel_mir49, novel_mir87, novel_mir40, novel_mir65, miR396h) exhibited higher expression levels in AZ than the other three groups (Fig. S6e and f).

To further understand the regulation of miRNAs and targets associated with the establishment of parasitic relationships, the expression levels of the selected miRNA targets were analyzed. These chosen target genes were predicted to be associated with biological regulation (CL9918.Contig2, CL14121.Contig1, Unigene14700, CL7316.Contig4, CL219.Contig5), membrane and organelle (Unigene4922, CL1529.Contig2, CL14340.Contig10, Unigene652, Unigene34515), and metabolism (Unigene7334, CL9246.Contig2, Unigene21854, Unigene8689, CL527.Contig67). The 15 target genes showed different relative expression levels in *M. savatieri* seedlings before and after parasitizing the host (Fig. S7). Five target genes (CL9918.Contig2, CL14340.Contig10, Unigene7334, CL9246.Contig2, CL527.Contig67) were strongly negatively correlated with the corresponding miRNAs. The expression level of *CL9918.Contig2* was up-regulated after establishment of the parasite-host association, while its corresponding miRNA (miR156_2) was down-regulated. In addition, six target genes (Unigene14700, CL14121.Contig1, CL219.Contig5, Unigene34515, Unigene21854, Unigene8689) were strongly positively correlated with the corresponding miRNAs. The expression level of *Unigene14700* was down-regulated after parasitizing the host, and its corresponding miRNA (novel_mir80) was also down-regulated.

### Correlation analysis of co-differentially expressed miRNAs and targets

**Fig. 9:**
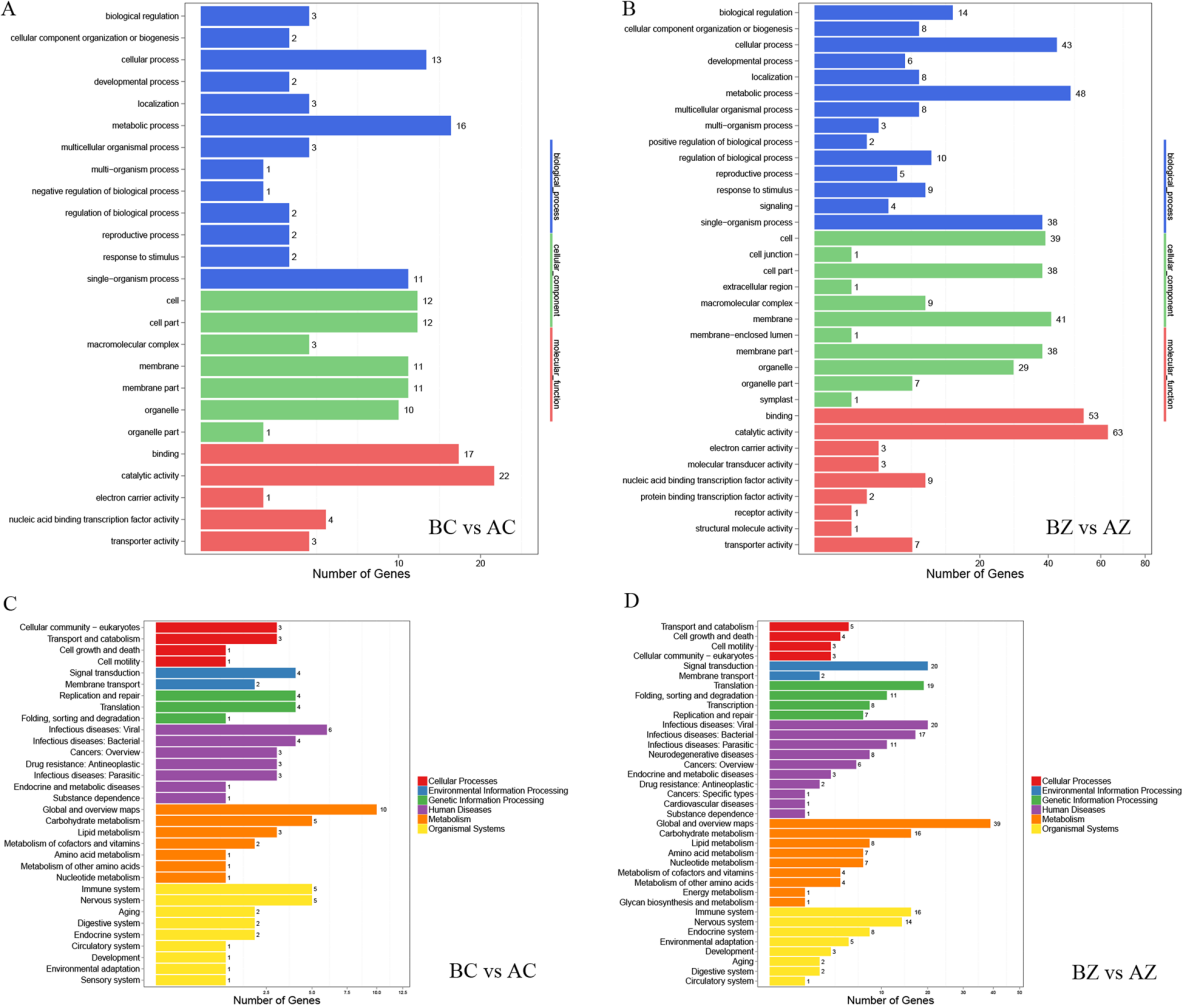
GO classification and KEGG pathway analysis of associated genes between miRNAs and target genes in *M. savatieri*. Panels a and b show the GO classification of associated genes in BC/AC (**a**) and BZ/AZ (**b**) comparisons. Panels c and d show the KEGG pathway analysis of associated genes in BC/AC (**c**) and BZ/AZ (**d**) comparisons. In Panels **a** and **b**, the number shown on the abscissa is obtained after taking the square root. BC, growing without a host for 8 WAS; BZ, growing with one *G. jasminoides* for 8 WAS; AC, growing without a host for 16 WAS; AZ, growing with one *G. jasminoides* for 16 WAS

In this research, the largest number of co-differentially expressed miRNAs and targets was observed in the BZ vs AZ comparison, including 58 negative correlations and 260 positive correlations. In the BC vs AC comparison, 30 miRNAs and 77 targets were co-differentially expressed (Table 5). To explore the biological functions and distributions of these associated genes between miRNAs and targets in *M. savatieri*, GO function annotations and KEGG pathway analysis were performed. The number of annotated genes for the same GO terms in the BZ vs AZ comparison was greater than the annotated genes in the BC vs AC comparison (Fig. 9a and b). Notably, “negative regulation of biological process” was detected only in the BC vs AC comparison, while “positive regulation of biological process” was detected only in the BZ vs AZ comparison. Moreover, the associated targets involved in “signaling”, “cell junction”, “protein binding transcription factor activity”, “membrane-enclosed lumen”, “molecular transducer activity”, “extracellular region” and “symplast” were specifically represented in the BZ vs AZ comparison (Fig. 9a and b). With regard to the KEGG pathway, the associated targets in the BZ vs AZ and BC vs AC comparisons were both significantly enriched in “cell growth and death”, “transport and catabolism”, “signal transduction”, “substance dependence”, “carbohydrate metabolism”, “development” and “environmental adaptation”, but the number of the associated genes for most of the same KEGG terms in the BZ vs AZ comparison was more than the number of the associated genes for most of the same KEGG terms in the BC vs AC comparison (Fig. 9c and d). Interestingly, “sensory system” was detected significantly enriched only in the BC vs AC comparison.

**Table 5:** Correlation results of co-differentially expressed miRNAs and target genes in *M. savatieri*

Diff group	Total			Negative			Positive		
	Co-diff numbers	miRNA numbers	Target numbers	Co-diff numbers	miRNA numbers	Target numbers	Co-diff numbers	miRNA numbers	Target numbers
AC/BZ	83	28	83	36	15	36	36	17	36
AZ/AC	0	0	0	0	0	0	0	0	0
BC/AC	78	30	77	21	11	21	36	21	35
BC/AZ	225	51	219	55	23	53	168	34	164
BC/BZ	20	14	19	1	1	1	19	14	18
BZ/AZ	320	53	310	58	22	57	260	39	251

BC, growing without a host for 8 WAS; BZ, growing with one *G. jasminoides* for 8 WAS; AC, growing without a host for 16 WAS; AZ, growing with one *G. jasminoides* for 16 WAS

**Fig. 10:**
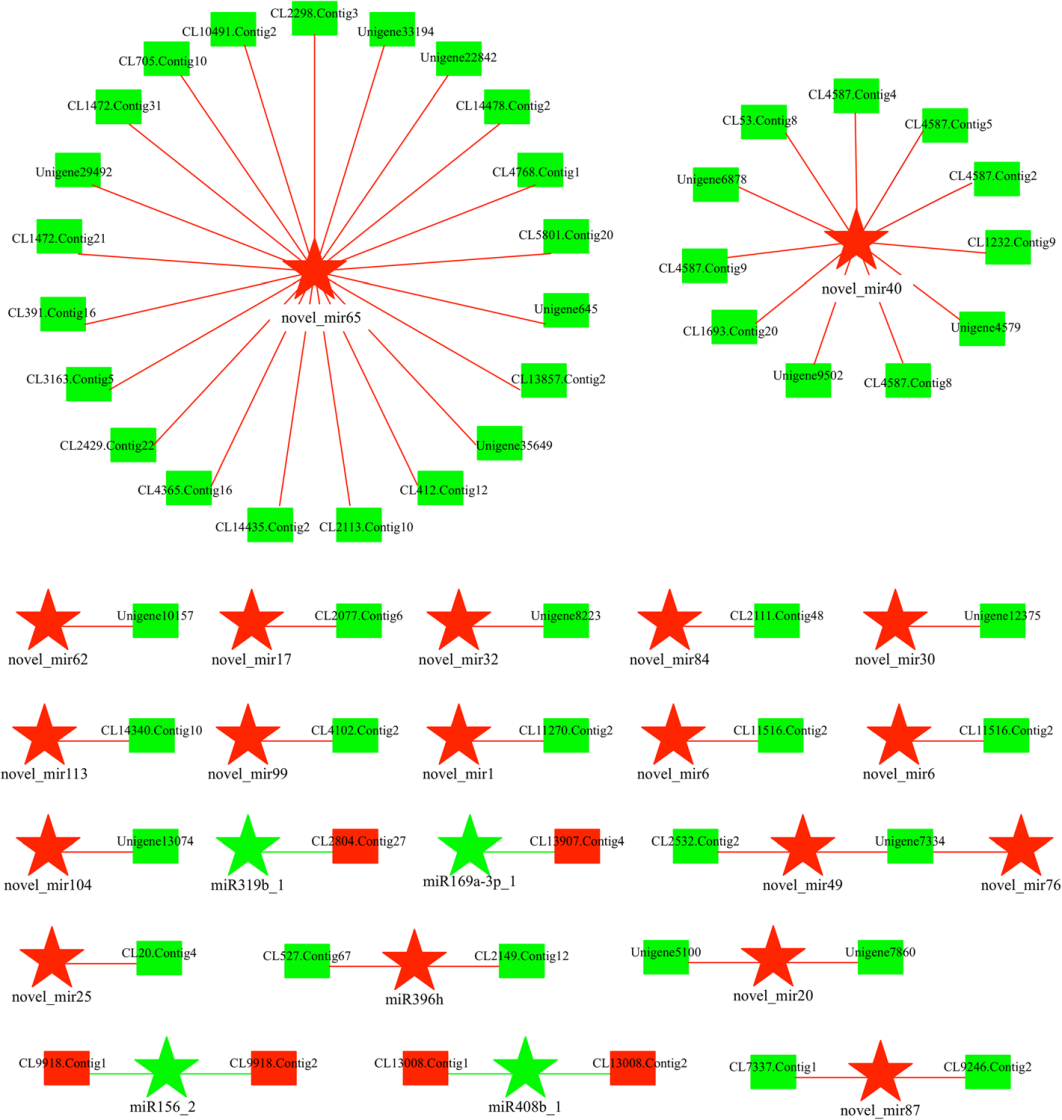
The interaction network of co-differentially negatively expressed miRNAs combined with mRNAs in the BZ/AZ comparison of *M. savatieri*. The stars represent miRNAs and rectangle nodes represent mRNAs. Red represents the up-regulated nodes, and green represents the down-regulated nodes. BZ, growing with one *G. jasminoides* for 8 WAS; AZ, growing with one *G. jasminoides* for 16 WAS

**Fig. 11:**
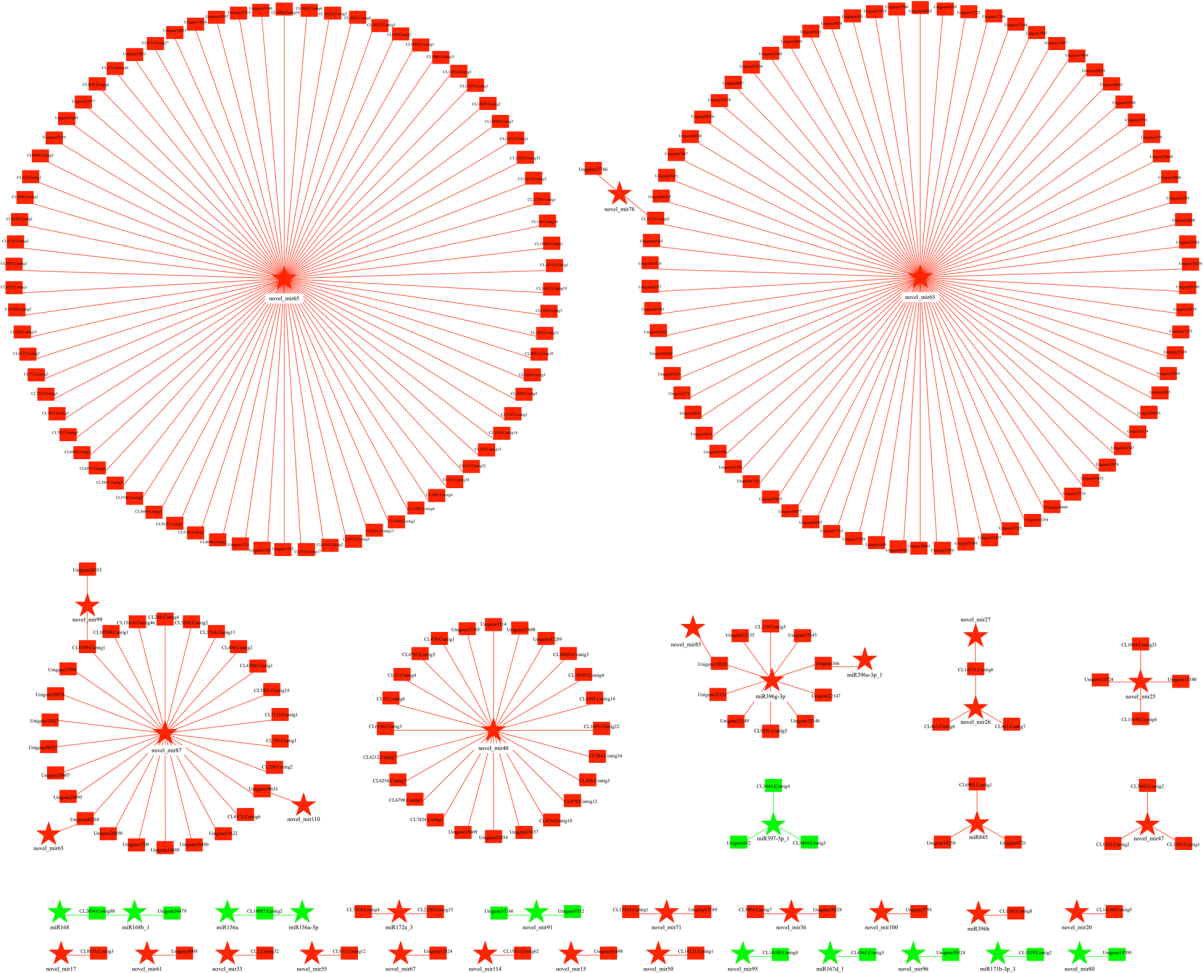
The interaction network of co-differentially positively expressed miRNAs combined with mRNAs in the BZ/AZ comparison of *M. savatieri*. The stars represent miRNAs and rectangle nodes represent mRNAs. Red represents the up-regulated nodes, and green represents the down-regulated nodes. BZ, growing with one *G. jasminoides* for 8 WAS; AZ, growing with one *G. jasminoides* for 16 WAS

Based on the mRNA-miRNA interaction network in the BZ/AZ comparison of *M. savatieri*, novel_mir65 contained the largest number of co-differentially expressed target genes, of which 21 and 147 associated genes targeted by novel_mir65 were negatively and positively expressed, respectively (Figs. 10, 11 and Additional file 6). Moreover, novel_mir40 had 11 co-differentially expressed target genes, with the second largest number of correlated miRNAs in the negatively regulated interaction network. Regarding the positively regulated interaction network, novel_mir87 and novel_mir40 had 26 and 24 co-differentially expressed target genes, being the second and third largest number of correlated miRNAs, respectively.

**Fig. 12:**
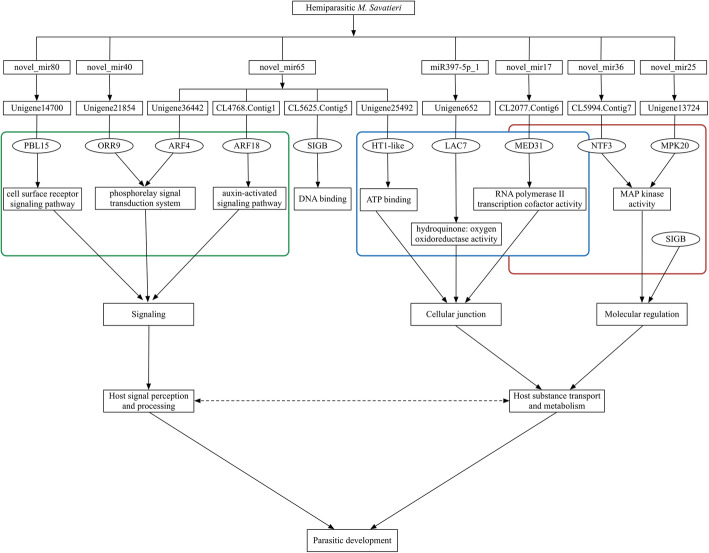
A putative miRNA regulatory diagram of the parasitic development of *M. savatieri*

Correlation analysis of transcriptome and miRNA data revealed several functional classifications specific to BZ/AZ. Therefore, miRNA-target gene pairs related to these functions were screened from the described interaction networks. Finally, 10 miRNA-target gene pairs were obtained from 7 miRNAs. The novel_mir65 had 4 target genes: CL4768. Contig1 was involved in the auxin-activated signaling pathway; Unigene 36,442 was involved in the phosphorelay signal transduction system; Unigene 25,492 was associated with ATP binding; and CL5625. Contig5 was related to DNA binding. The novel_mir80 had a target gene, Unigene14700, involved in the cell surface receptor signaling pathway; novel_mir40 had a target gene (Unigene 21,854) related to the phosphorelay signal transduction system; miR397-5p_1 had a target gene (Unigene 652) related to hydroquinone: oxygen oxidoreductase activity; novel_mir17 had a target gene (CL2077. Contig6) related to RNA polymerase II transcription cofactor activity; the target genes of novel_mir36 (CL5994. Contig7) and novel_mir25 (Unigene 13,724) were associated with MAP kinase activity (Fig. 12). These seven miRNAs may affect the parasitic development of *M. savatieri* by regulating the related genes of signaling, cellular junctions, and molecular regulation.

## Discussion

*M. savatieri* is a medically important parasite, and much attention has been given to artificial cultivation because of its sharply declining wild resources. The essence of life habits in parasites is to establish parasitic relationships with the host [23]. In recent decades, research on the establishment of parasitic relationship has revealed its role in promoting the growth and development of some hemiparasitic plants including *Pedicularis rex* and *Thesium chinense* [24, 25]. In our recent study, the growth and development of *M. savatieri* was significantly promoted after parasitizing the host (Fig. 1a). However, to date, there has been no study on the underlying molecular mechanisms, and little is known about the characteristics of related endogenous hormones. This study is the first to explore the changes in the transcriptome and small RNA before and after establishing parasitic relationships with the host.

Phytohormones are important factors regulating plant growth and development. By analyzing the KEGG pathway, many DEGs were found to be enriched in “plant hormone signal transduction” (Fig. 3e and f). The present study showed that levels of auxins such as IAA, ME-IAA, and ICA showed a significant decrease with *M. savatieri* development, independent of establishing the parasite-host association (Fig. 1b and c). Similarly, a downtrend of IAA content was detected in *Cuscuta japonica* during its parasitization [11]. Although JA and SA levels in *M. savatieri* grown without the host increased significantly with development, contrary variation was observed in seedlings grown with the host (Fig. 1e and f). JA is reported to regulate plant growth and stress responses [26], and SA is found to mediate stress tolerance [27]. However, the functions of JAs and SA in parasitic plants are still unknown. We speculate that JAs and SA may play a crucial role in stress-acclimation signaling, and the decreased JA and SA levels in attached *M. savatieri* seedlings may be related to a reduced demand for protection. Increasing ABA levels in attached hemiparasites have been reported in *Rhinanthus minor* [28]. ABA levels in *M. savatieri* followed a similar trend, increasing significantly after parasitizing the host compared with seedlings grown with the host for 8 WAS (Fig. 1f). The high levels of ABA in attached hemiparasites may be due to the increase in self-synthesized ABA and ABA obtained from the host [29]. Surprisingly, *M. savatieri* grown for 16 WAS had approximately 3.7 times more ABA than *M. savatieri* grown for 8 WAS even when growing without the host. It is possible that parasites that have been unattached for a long time were susceptible to abiotic stress, thus stimulating the increase in ABA, which is a plant drought-related hormone. These results provide a clue for improving the growth of beneficial parasites by exogenous application of plant growth regulators, although further evidence needs to be obtained to verify the effects.

In this study, most DEGs associated with “starch and sucrose metabolism” were enriched in the comparisons of *M. savatieri* plants with established parasitic relationships (Fig. 3e and f). Moreover, associated genes between miRNAs and target genes were also involved in “carbohydrate metabolism” of *M. savatieri* (Fig. 9c and d). Similarly, unigenes associated with carbohydrate, starch and sucrose metabolism have been identified in the development of the parasite *Arceuthobium sichuanense* at the transcriptional level [30]. Combined with the results of our research, these data collectively suggest that the metabolism of carbohydrates, starch and sucrose could have an essential role in the development of *M. savatieri* after establishing parasitic relationships with the host. In our recent research, the observation that the growth of *M. savatieri* in the absence of a host plant was increased under the condition of sufficient nitrogen was first reported [31]. Interestingly, the present study showed that the DEGs significantly enriched in “nitrogen metabolism” and “photosynthesis” were observed only in the development of *M. savatieri* grown without the host (Fig. 3a). Considering that the increasing nitrogen supply stimulated the photosynthetic rates of *Striga hermonthica* [32], and the retained photosynthetic capacity in *M. savatieri*, we speculated that nitrogen metabolism may be closely related to photosynthesis during autotrophic growth, which might suggest a survival strategy for root hemiparasites in the absence of the host. Furthermore, the DEGs and DEmiR targets involved in “carbon fixation in photosynthetic organisms” were enriched only in the development of *M. savatieri*, which growing without *G. jasminoides* (Figs. 3a, 8a). The findings that most carbon used by the parasites came from the host may partially explain these results [33], suggesting that *M. savatieri* largely depended on the photosynthesis of carbon before attaching to the host.

Based on transcriptome and small RNA sequencing in *M. savatieri*, the correlation analysis demonstrated that most co-differentially expressed miRNAs and targets were represented in the comparisons of plants with established parasitic relationships (Table 5). In previous studies, mRNAs were found to move bidirectionally between parasites and hosts [16], and parasite miRNA-targeted host mRNAs act as trans-species regulators during parasitism [22], implying that genetic molecule exchange may also take place in *M. savatieri*-host plant interactions, and most miRNAs and targets play significant roles in the post-parasitization stage. Notably, neither positive nor negative correlations were found in the co-differentially expressed miRNAs and targets in the AC vs AZ comparison (Table 5). The results are not in line with our expectations. Further transcriptome and small RNA sequencing of the host plant will be useful to understand the mechanisms underlying the results and verify *M. savatieri*-host plant interactions. In addition, the associated targets participating in “cell junction”, “extracellular region”, “membrane-enclosed lumen” and “symplast” were differentially expressed in *M. savatieri* plants before and after establishing parasitic relationships with *G. jasminoides* (Fig. 9a and b). The symplastic or apoplastic transport of nutrient acquisition from the host plant by the parasite has been discussed in recent years [34]. The analysis of RNA-seq and sRNA-seq demonstrated that the transport mechanism of *M. savatieri*-acquired nutrients from the host plant included symplast in addition to apoplast. The result was similar to the finding reported for *C. pentagona* [14].

There were 162 differentially expressed known and novel miRNAs identified in *M. savatieri* before and after establishing parasitic relationships with the host (Fig. 7a); however, only 53 DEmiRs led their target genes to exhibit significantly different expression (Figs. 10 and 11). Considering the role of phased small interfering RNA (siRNA) in growth regulation [35], we speculated that siRNA may also act as a regulator in the gene expression of *M. savatieri* development. The roles of miRNAs in regulating plant development have been studied extensively. In *Arabidopsis*, miR167 regulates the expression pattern of the *ARF6* and *ARF8* genes and is essential for the fertility of ovules and anthers [36]. Overexpression of miR396 results in a reduction of growth-regulating factor expression and the cell number in leaves of *A. thaliana* [37]. In this mRNA-miRNA interaction network, the known miRNAs belonging to miR167, miR396 and miR845 families, and their targets were co-differentially expressed in the roots of *M. savatieri* before and after establishing parasitic relationships (Figs. 10 and 11). Of these miRNAs, most known miRNAs were conserved among plant species. However, miR845 has not been commonly reported in plants, suggesting that some miRNAs may be specific to some species. Moreover, miR396 was also one of the most numerous families in *M. savatieri* (Fig. 6c), which suggested that miR396 might have a fundamental role in the processes of parasite development. However, based on the mRNA-miRNA interaction network, most co-differentially expressed genes were targeted by some novel miRNAs, including novel_mir65, novel_mir40 and novel_mir87 (Figs. 10 and 11). Therefore, further molecular experiments should be conducted to explore their roles and mechanisms during parasitism of *M. savatieri*.

## Conclusions

Establishing parasitic relationships with the host decreased the levels of IAA and JA and increased the ABA content in *M. savatieri*. Our study provided the first transcriptome and small RNA resources for further investigations of the responsible molecular mechanisms of enhanced growth in *M. savatieri* after establishing parasitic relationship. Using high-throughput sequencing, transcriptome data consisting of 167,941 unigenes were obtained from *M. savatieri* roots, and 46,424 DEGs were detected in the comparison between *M. savatieri* with the established parasitic relationship and without the established parasitic relationship. Analysis of small RNA data obtained 128 novel miRNAs and 110 known miRNAs, and 198 miRNAs were significantly differentially expressed in *M. savatieri*. The DEGs and DEmiRs related to growth and development in *M. savatieri* were revealed by the GO and KEGG pathway enrichment analysis. Moreover, correlation analysis of mRNA and miRNA revealed that 10 miRNA-target pairs from novel_mir65, novel_mir40, novel_mir80, miR397-5p_1, novel_mir36, novel_mir25 and novel_mir17 may have vital roles in regulating the parasitic development of *M. savatieri*. The data generated by this study provided abundant information and gene and miRNA sequences to accelerate the molecular and genetic studies in *M. savatieri*. In the future, research is needed to know the exact mechanisms by which the establishment of parasite-host associations regulates growth and physiological processes.

## Methods

### Plant materials and growth conditions

*M. savatieri* seed capsules at fully mature were collected from the experimental base, located at Yongfeng County, Jiangxi Province, of the Huizhou Jiuhui Pharmaceutical Co., Ltd., in May 2017 (the collection was permitted by Huizhou Jiuhui Pharmaceutical Co., Ltd.), and then stored in paper bags at 4 °C until required. *M. savatieri* was identified and authenticated by Professor Qiaosheng Guo, Nanjing Agricultural University. The voucher specimen was deposited in the Institute of Chinese Medicinal Materials, Nanjing Agricultural University (Nanjing, China). Due to *Gardenia jasminoides* E. (Rubiaceae) was widely distributed in the plant community of *M. savatieri* [3] and could be parasitized by potted *M. savatieri* [7], it was used as a host in this experiment. *M. savatieri* seedlings were grown in the presence or absence of one *G. jasminoides* plant. On March 19, 2018, after washing with tap water, we transplanted the commercially available *G. jasminoides* plants of about 15 cm in height (purchased from Lingshan Yufeng Guomiao Co., Ltd) into the pot filled with 770 g of fine sand and nutritive soil (2:1, v/v), one *G. jasminoides* plant per pot. In order to promote seed germination, the 800 mg·L^− 1^ gibberellin solution was used to soak *M. savatieri* seeds for 24 h at room temperature [38]. These seeds were sown on April 17, 2018, and each pot contained 50 seeds. Thinning was carried out at 4 weeks after sowing (WAS), and 10 seedlings of about 5 mm in height were kept in each pot [7].

All plants were cultivated in a natural night/day greenhouse at the Institute of Chinese Medicinal Materials, Nanjing Agricultural University. This experiment was conducted for 20 weeks from spring (March, 2018) to summer (August, 2018). After thinning (from May to August), the greenhouse was covered with a 50% Sun-Block shade net to make the light condition similar to that of *M. savatieri* habitats. During the whole experiment, the pots were watered with tap water every day to reach field capacity and placed fully at random, and were rerandomized every 2 weeks to minimize the effect of position.

### Harvest and sampling

Two harvests were conducted to examine the differences between *M. savatieri* grown in the presence or absence of *G. jasminoides* at two key developmental phases of the parasitic plants [23]. One at 8 WAS (early seedling phase, before establishing parasitic relationship with the host), and the other at 16 WAS (late seedling phase, after establishing parasitic relationship with the host). The obvious enhancement of hemiparasite growth confirmed the successful establishment of *M. savatieri*-*G. jasminoides* association [39]. The experiment included four different groups. BC, *M. savatieri* that had grown without *G. jasminoides* for 8 WAS; BZ, *M. savatieri* that had grown with *G. jasminoides* for 8 WAS; AC, *M. savatieri* that had grown without *G. jasminoides* for 16 WAS; AZ, *M. savatieri* that had grown with *G. jasminoides* for 16 WAS. For analysis of plant endogenous hormones, seedling samples from each group were collected with three replicates and weighed for further measurement. For analysis of transcriptome and small RNA, *M. savatieri* roots collected from each group were quickly frozen in liquid nitrogen and then stored at − 80 °C until required. There were three replicates for transcriptome and small RNA analysis.

### Endogenous hormone measurement

The extraction, purification and measurement of IAA, ME-IAA, IC), tZ, cZ, JA, H2JA, jasmonoyl-(L)-isoleucine (JA-ILE), SA and ABA were performed as previously reported [40]. Briefly, 1 g of fresh weight of *M. savatieri* samples was extracted with 10 mL methanol/water/formic acid (15:4:1, v/v/v) at 4 °C. The LC-ESI-MS/MS system (MS, Applied Biosystems 6500 Triple Quadrupole; HPLC, Shim-pack UFLC SHIMADZU CBM30A system) was utilized to analyze the generated extracts, with the ESI-triple quadrupole-linear ion trap (Q TRAP)-MS alternatively connected to the effluent. In addition, the API 6500 Q TRAP LC/MS/MS System controlled by Analyst 1.6 software (AB Sciex) was equipped with the ESI Turbo Ion-Spray interface and operated in positive ion mode.

### Total RNA extraction, library construction and sequencing

Total RNA extraction of the root tissues in *M. savatieri* was performed using the TRIzol reagent kit (Invitrogen, USA) based on the manufacturer’s guidance. The Nanodrop 8000 spectrophotometer (Thermo Fisher Scientific, USA) and Agilent 2100 Bioanalyzer (Agilent Technologies, USA) were used to evaluate the quantity and quality of these total RNAs, respectively. The qualified total RNA was ready for transcriptome and small RNA sequencing using the BGISEQ-500 platform. A total of 12 mRNA and sRNA libraries were constructed with three biological replicates for each treatment. Sequencing, data processing, gene functional annotation, and miRNA identification were conducted by BGI (Shenzhen, China). Both RNA-seq and small RNA-seq data have been deposited in the NCBI SRA database, with BioProject IDs PRJNA600790 and PRJNA601043, respectively. Trinity software (parameter: --min_contig_length 150 --CPU 8 --min_kmer_cov 3 --min_glue 3 --bfly_opts ‘-V 5 --edge-thr = 0.1 --stderr’) was adopted to de novo assembly of the filtered clean reads [41]. In addition, we performed BUSCO with default parameters to check the quality of the assembled transcripts [42].

### Transcriptome and small RNA analysis

The known miRNAs were identified by matching the sequences to the known mature miRNAs in the miRBase. Based on the characteristic hairpin secondary structure of the miRNA precursor, miRA [43] was adopted to predict novel miRNA using default parameters (cluster_min_reads = 10; min_precursor_length = 50; max_mfe_per_nt = − 0.2; min_duplex_length = 18; max_duplex_length = 30; allow_three_mismatches = 1). The DESeq R package (1.18.0) was used for analysis of the differential expression of pairs. To control the false discovery rate, the approach of Benjamini and Hochberg was adopted to adjust the *P*-value. The conditions used to screen the DEGs and DEmiRs were a fold change ≥2.00 and an adjusted *P*-value < 0.05 [44]. GO and KEGG enrichment analyses of the DEGs and target genes of DEmiRs were implemented based on the hypergeometric model [45]. To obtain more accurate results, both psRobot (parameter: -gl 17 -p 8 -gn 1) [46] and TargetFinder (parameter: -c 4) [47] were adopted to predict the target genes of miRNAs, and the corresponding intersection was selected as the final result.

### Analysis of DEG network

To perform the analysis of network relations, the DEGs were compared with the STRING database by DIAMOND, and the interaction between genes was obtained by homology with known proteins. There is a score value for the interaction between genes, which indicates the reliability of the interaction. From 150 to 1000, the higher the score, the more reliable the interaction relationship. In our analysis, the network relations with score ≥ 300 were selected for building the DEG network.

### Correlation analysis of mRNA and miRNA

To further understand all the possible interactions between mRNA and miRNA (negative and positive correlations of mRNA-miRNA expression), an mRNA-miRNA regulatory network was built using the in-house R script. In brief, to integrate the DEmiRs with DEGs, matched mRNA and miRNA sequencing data for all samples were normalized, and then the expression profiles of mRNA and miRNA, the information for the miRNA target, and sample categories were integrated.

### Quantitative real-time PCR analysis

In the experiment, 14 genes, 15 miRNAs and target genes associated with “cell part”, “plant hormone signal transduction”, and “metabolism” were selected to analyze the expression levels by qRT-PCR. Based on the instructions of the manufacturer, the TaKaRa MiniBEST Plant RNA Extraction Kit (TaKaRa, China) was adopted to extract the total RNA from BC, BZ, AC and AZ. The gDNA Eraser Perfect Real Time and PrimeScript™ RT reagent Kit (TaKaRa, China) were adopted to synthesize cDNA used for the qRT-PCR analysis of genes and miRNA targets. The miRNA expression was validated by RNA-tailing, and cDNA used for the qRT-PCR analysis of miRNAs was synthesized using the Mir-X™ miRNA First-Strand Synthesis and TB Green™ qRT-PCR Kit (TaKaRa, China). The amplified reaction was conducted with the Step One Real-Time system (Applied Biosystems, USA) based on a previous report [48]. Three biological replicates were applied. All primer sequences are given in Tables S2, S3 and S4. For the genes and miRNA targets, the relative expression levels were normalized against the geometric average of the *GAPDH* and *actin* genes [49]. For the miRNAs, U6 snRNA served as the internal control, and the analysis was carried out based on the 2^−ΔΔCT^ method [50].

### Statistical analysis

IBM Statistical Product and Service Solutions (SPSS) 22.0 software (IBM Corporation, USA) was used to perform the statistical analysis, and the means were compared by Duncan’s multiple range test (*P* < 0.05).

## Supplementary Information

**Additional file 1: Fig. S1.** Unigene sequence length distribution in the transcriptome of *M. savatieri*. **Fig. S2.** GO classification of all unigenes in *M. savatieri*. **Fig. S3.** Heatmap clustering and qRT-PCR analysis of DEGs in *M. savatieri*. (a) Transcript abundances of DEGs related to starch and sucrose metabolism. (b) Relative expression levels of DEGs related to starch and sucrose metabolism. (c) Transcript abundances of DEGs related to hormone signal transduction. (d) Relative expression levels of DEGs related to hormone signal transduction. (e) Transcript abundances of DEGs related to cell part. (f) Relative expression levels of DEGs related to cell part. The error bars of qRT-PCR data indicate the standard deviations of the three replicate determinations. **Fig. S4.** Nucleotide length distribution of small RNAs in *M. savatieri*. **Fig. S5.** The first base distribution of known miRNAs (a) and novel miRNAs (b) in *M. savatieri*. In the bar graphs, the X-axis represents the length of miRNA, the number shown on the bar represents the number of miRNA at this length. **Fig. S6.** Heatmap clustering and qRT-PCR analysis of miRNAs in *M. savatieri*. (a) Sequencing abundances of miRNAs related to biological regulation. (b) Relative expression levels of miRNAs related to biological regulation. (c) Sequencing abundances of miRNAs related to membrane and organelle. (d) Relative expression levels of miRNAs related to membrane and organelle. (e) Sequencing abundances of miRNAs related to metabolism. (f) Relative expression levels of miRNAs related to metabolism. The error bars of qRT-PCR data indicate the standard deviations of the three replicate determinations. **Fig. S7.** qRT-PCR analysis of miRNA target genes in *M. savatieri*. The error bars of qRT-PCR data indicate the standard deviations of the three replicate determinations. **Table S1.** Transcription factor families of the DEGs related to the biological processes in the BZ/AZ comparison of *M. savatieri*. **Table S2.** Specific primers of differentially expressed genes for qRT-PCR validation. **Table S3.** Specific primers of differentially expressed miRNAs for qRT-PCR validation. **Table S4.** Specific primers of target genes of differentially expressed miRNAs for qRT-PCR validation.

**Additional file 2.** List of DEGs among different comparisons.

**Additional file 3.** GO classification of DEGs among different comparisons.

**Additional file 4.** List of DEmiRs among different comparisons.

**Additional file 5.** KEGG pathway of DEmiR target genes among different comparisons.

**Additional file 6.** Co-differentially expressed miRNAs and targets in the BC/AC and BZ/AZ comparisons.

## Data Availability

The RNA-seq read data analyzed during the current study are available in the NCBI SRA database, https://www.ncbi.nlm.nih.gov/sra/PRJNA600790 (accession BioProject number: PRJNA600790). The Small RNA-seq read data analyzed during the current study are available in the NCBI SRA database, https://www.ncbi.nlm.nih.gov/sra/PRJNA601043 (accession BioProject number: PRJNA601043).

## Abbreviations

RNA-seq: RNA sequencing
DEGs: Differentially expressed genes
DEmiRs: Differentially expressed miRNAs
NR: Non-redundant protein database
NT: Nucleotide sequence database
GO: Gene Ontology
KEGG: Kyoto Encyclopedia of Genes and Genomes
sRNAs: Small RNAs
WAS: Weeks after sowing
IAA: Indole-3-acetic acid
ME-IAA: Methyl indole-3-acetate
ICA: Indole-3-carboxaldehyde
tZ: Trans-zeatin
cZ: Cis-zeatin
JA: Jasmonic acid
H2JA: Dihydrojasmonic acid
JA-ILE: Jasmonoyl-(L)-isoleucine
SA: Salicylic acid
ABA: Abscisic acid
qRT-PCR: Quantitative real-time PCR
