# Supplementary material for: Integrated analyses of the transcriptome and small RNA of the hemiparasitic plant Monochasma savatieri before and after establishment of parasite-host association
Source: BMC Plant Biol. 2021 Feb 10;21:90. doi: 10.1186/s12870-021-02861-6 (PMC7877053; doi:10.1186/s12870-021-02861-6)
Supplement: Supplementary file 1 — Additional file 1: Fig. S1. Unigene sequence length distribution in the transcriptome of M. savatieri. Fig. S2. GO classification of all unigenes in M. savatieri. Fig. S3. Heatmap clustering and qRT-PCR analysis of DEGs in M. savatieri. (a) Transcript abundances of DEGs related to starch and sucrose metabolism. (b) Relative expression levels of DEGs related to starch and sucrose metabolism. (c) Transcript abundances of DEGs related to hormone signal transduction. (d) Relative expression levels of DEGs related to hormone signal transduction. (e) Transcript abundances of DEGs related to cell part. (f) Relative expression levels of DEGs related to cell part. The error bars of qRT-PCR data indicate the standard deviations of the three replicate determinations. Fig. S4. Nucleotide length distribution of small RNAs in M. savatieri. Fig. S5. The first base distribution of known miRNAs (a) and novel miRNAs (b) in M. savatieri. In the bar graphs, the X-axis represents the length of miRNA, the number shown on the bar represents the number of miRNA at this length. Fig. S6. Heatmap clustering and qRT-PCR analysis of miRNAs in M. savatieri. (a) Sequencing abundances of miRNAs related to biological regulation. (b) Relative expression levels of miRNAs related to biological regulation. (c) Sequencing abundances of miRNAs related to membrane and organelle. (d) Relative expression levels of miRNAs related to membrane and organelle. (e) Sequencing abundances of miRNAs related to metabolism. (f) Relative expression levels of miRNAs related to metabolism. The error bars of qRT-PCR data indicate the standard deviations of the three replicate determinations. Fig. S7. qRT-PCR analysis of miRNA target genes in M. savatieri. The error bars of qRT-PCR data indicate the standard deviations of the three replicate determinations. Table S1. Transcription factor families of the DEGs related to the biological processes in the BZ/AZ comparison of M. savatieri. Table S2. Specific primers of different [file 12870_2021_2861_MOESM1_ESM.zip › Additional file 1.docx]

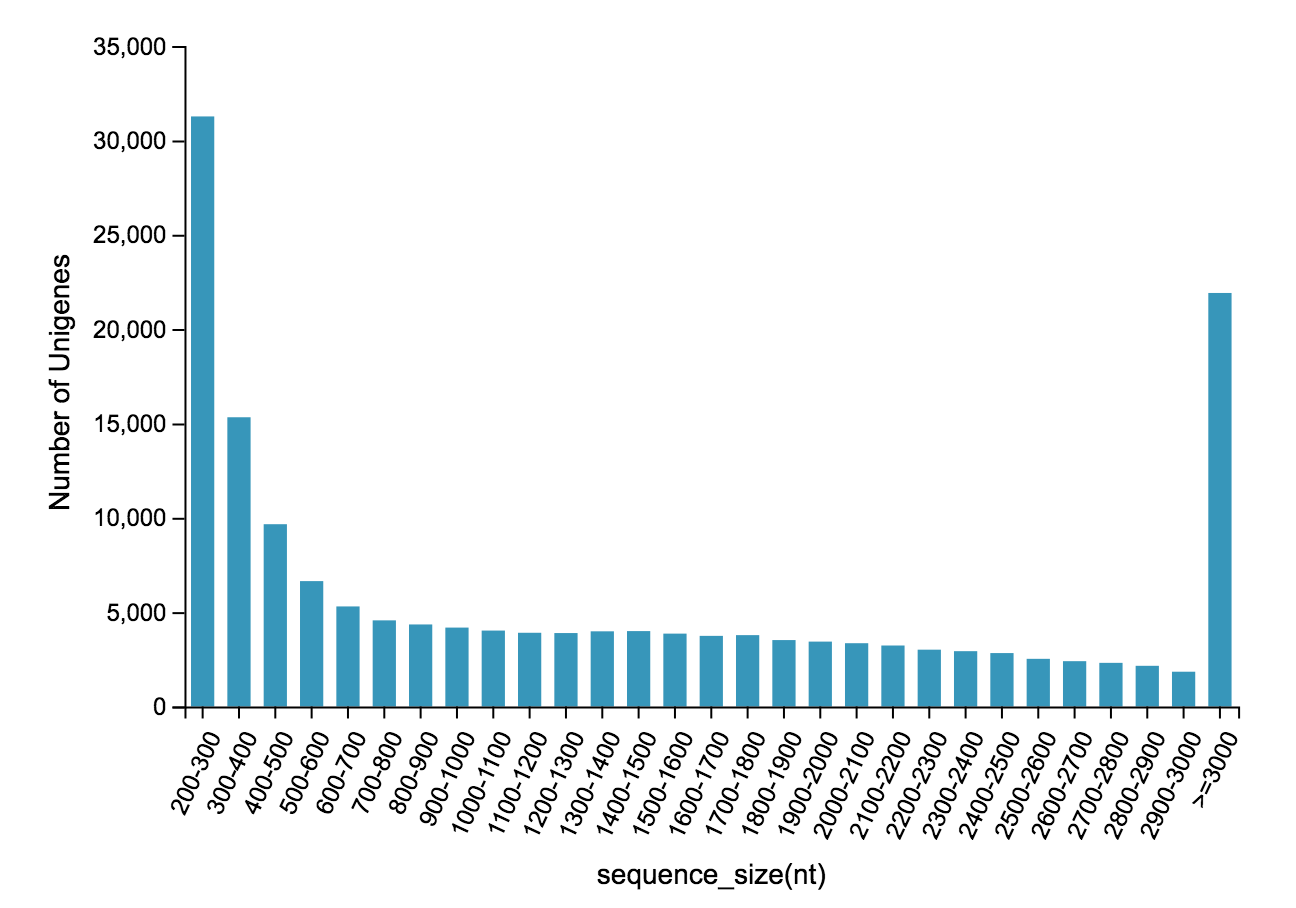


**Figure S1. Unigene sequence length distribution in the transcriptome of *M. savatieri*.**


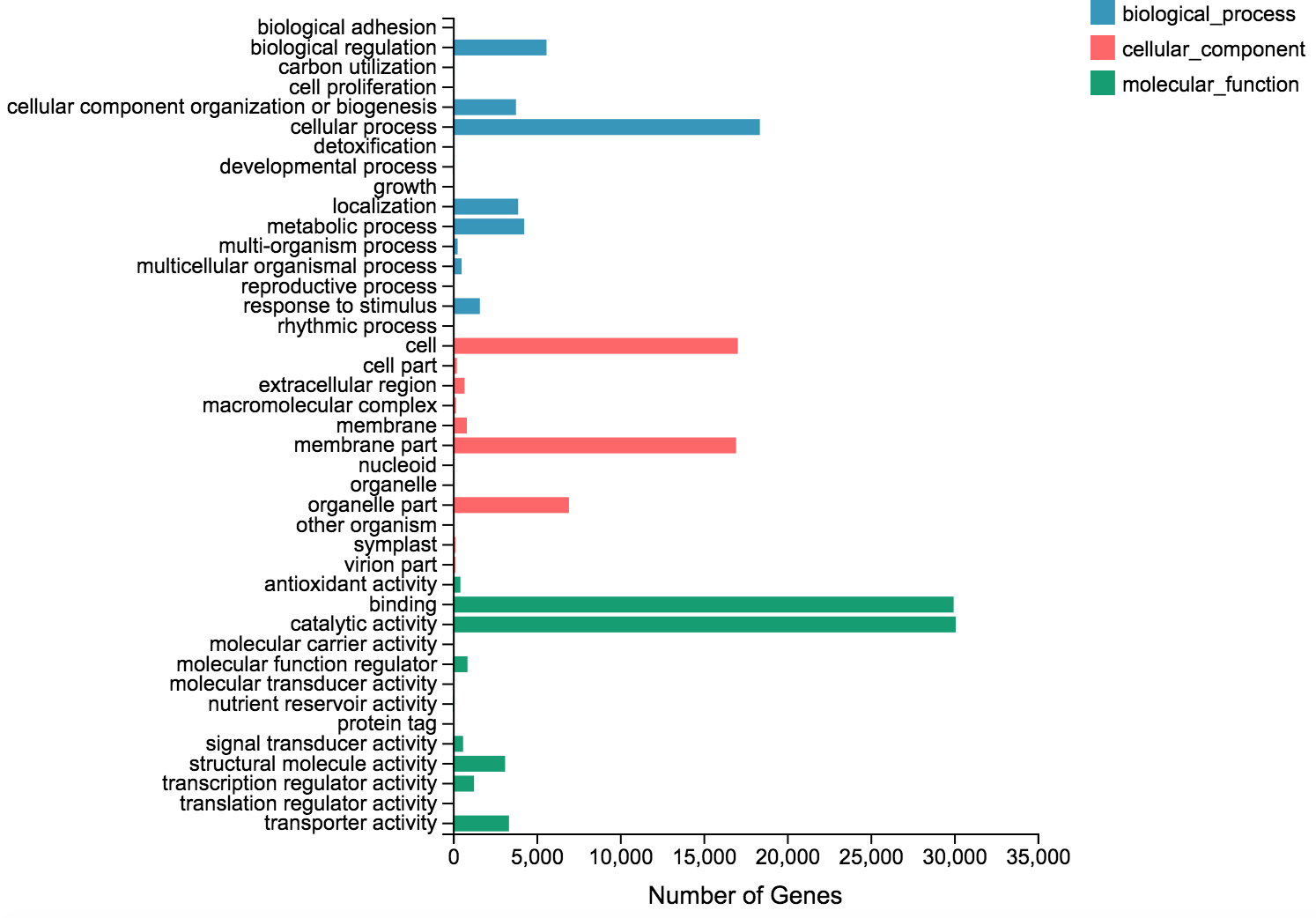


**Figure S2. GO classification of all unigenes in *M. savatieri*.**


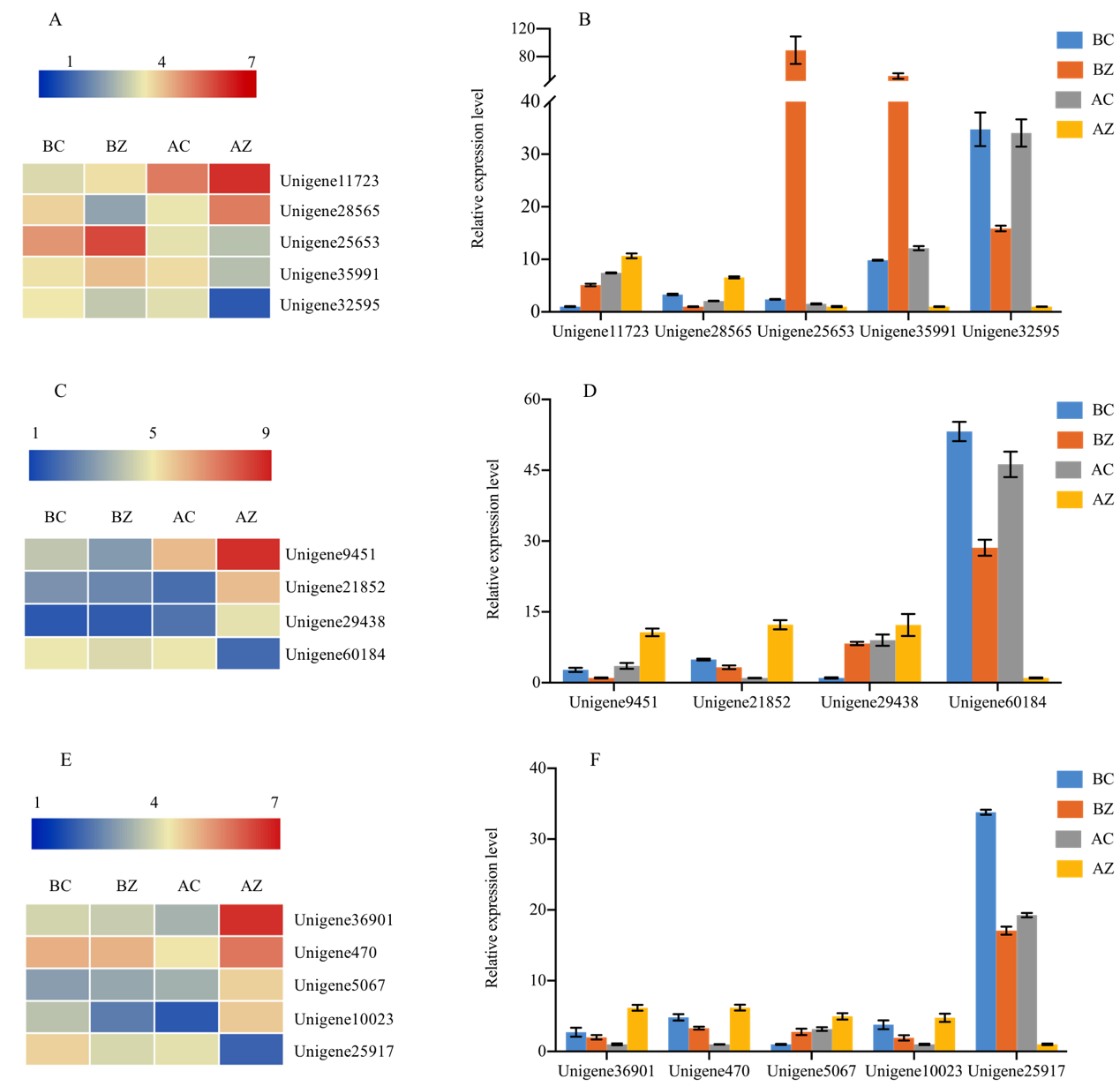


**Figure S3. Heatmap clustering and qRT-PCR analysis of DEGs in *M. savatieri*.** (a) Transcript abundances of DEGs related to starch and sucrose metabolism. (b) Relative expression levels of DEGs related to starch and sucrose metabolism. (c) Transcript abundances of DEGs related to hormone signal transduction. (d) Relative expression levels of DEGs related to hormone signal transduction. (e) Transcript abundances of DEGs related to cell part. (f) Relative expression levels of DEGs related to cell part. The error bars of qRT-PCR data indicate the standard deviations of the three replicate determinations.


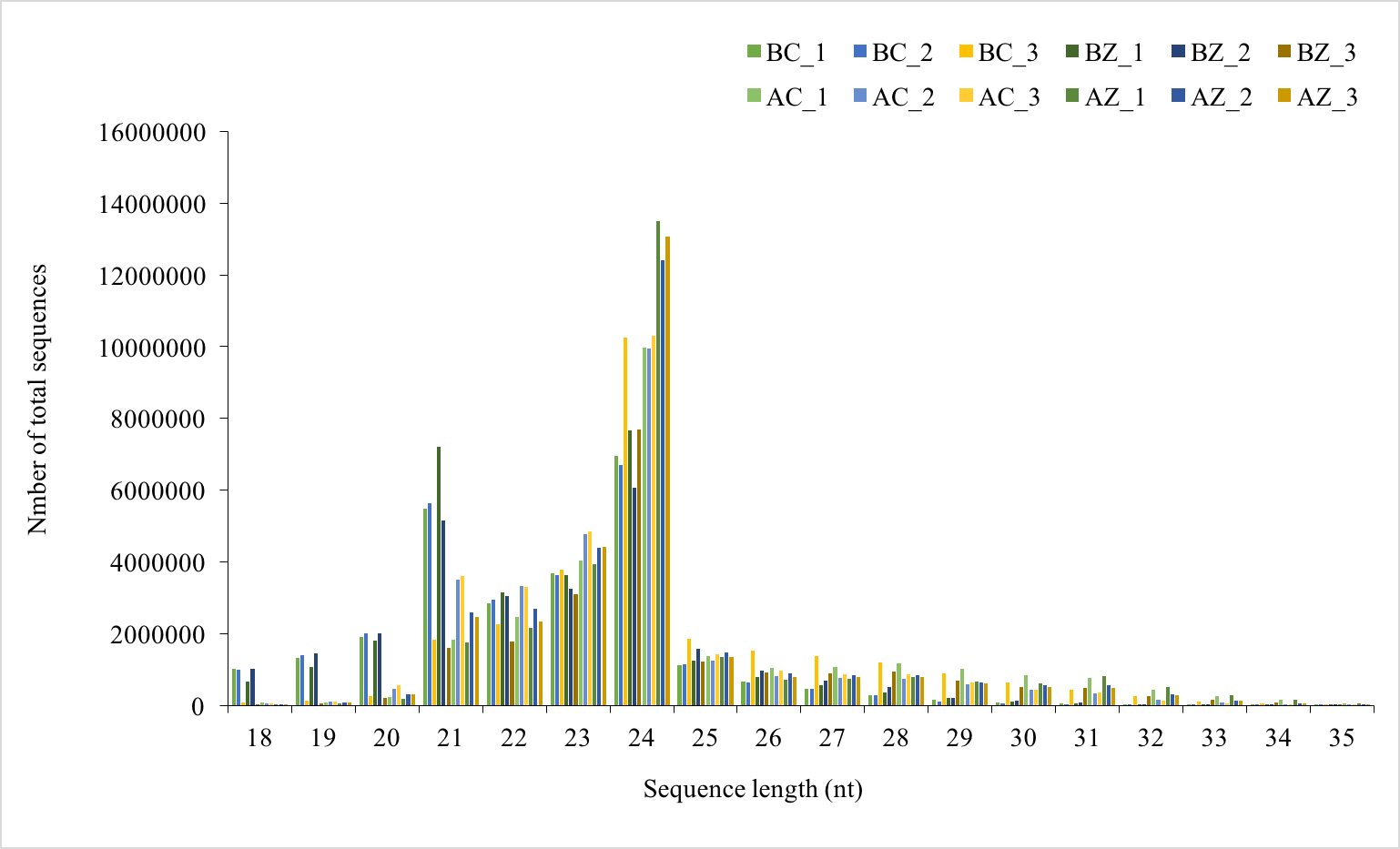


**Figure S4. Nucleotide length distribution of small RNAs in *M. savatieri*.**

**Figure S5. The first base distribution of known miRNAs (a) and novel miRNAs (b) in *M. savatieri*.** In the bar graphs, the X-axis represents the length of miRNA, the number shown on the bar represents the number of miRNA at this length.


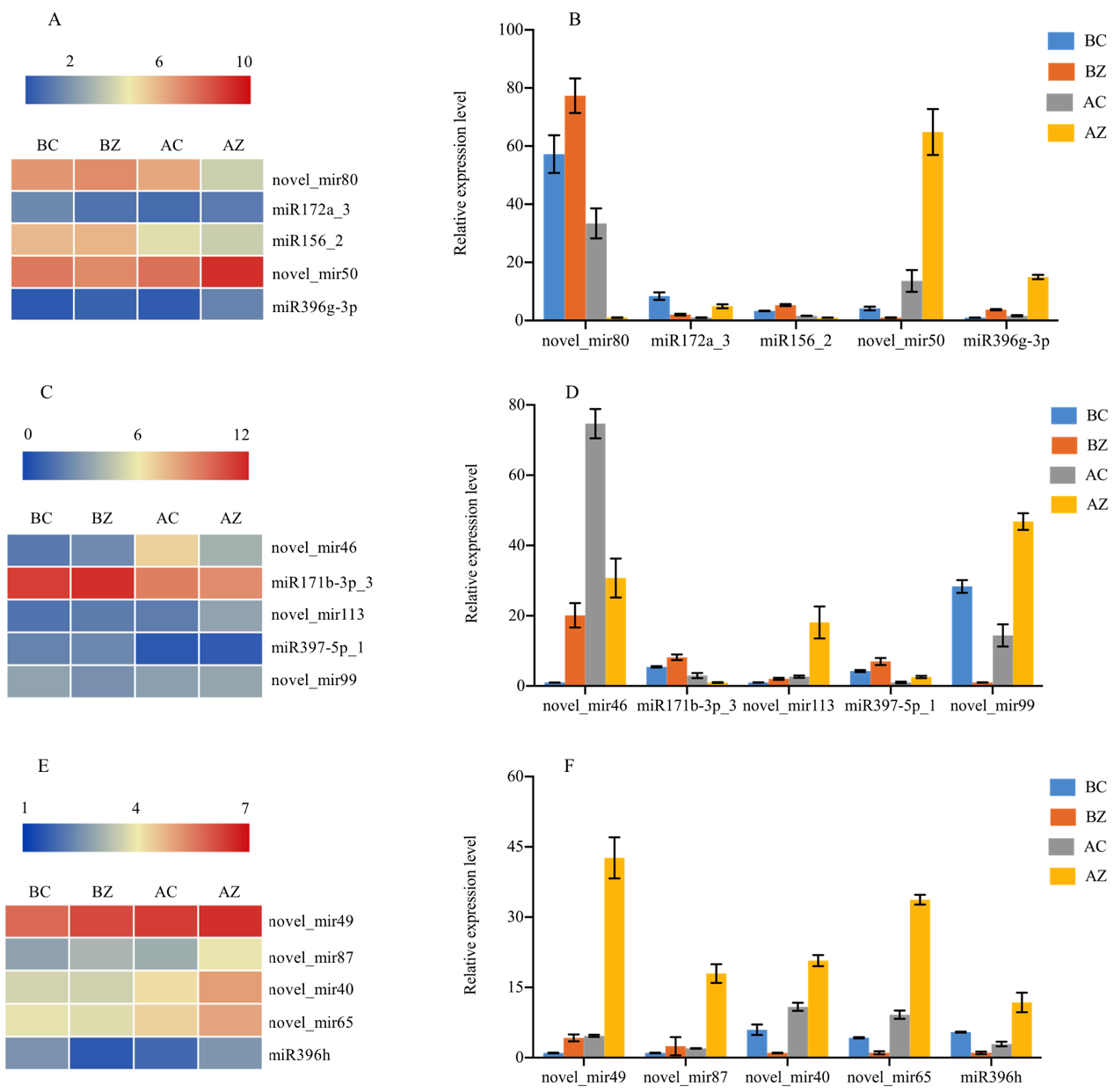


**Figure S6. Heatmap clustering and qRT-PCR analysis of miRNAs in *M. savatieri*.** (a) Sequencing abundances of miRNAs related to biological regulation. (b) Relative expression levels of miRNAs related to biological regulation. (c) Sequencing abundances of miRNAs related to membrane and organelle. (d) Relative expression levels of miRNAs related to membrane and organelle. (e) Sequencing abundances of miRNAs related to metabolism. (f) Relative expression levels of miRNAs related to metabolism. The error bars of qRT-PCR data indicate the standard deviations of the three replicate determinations.


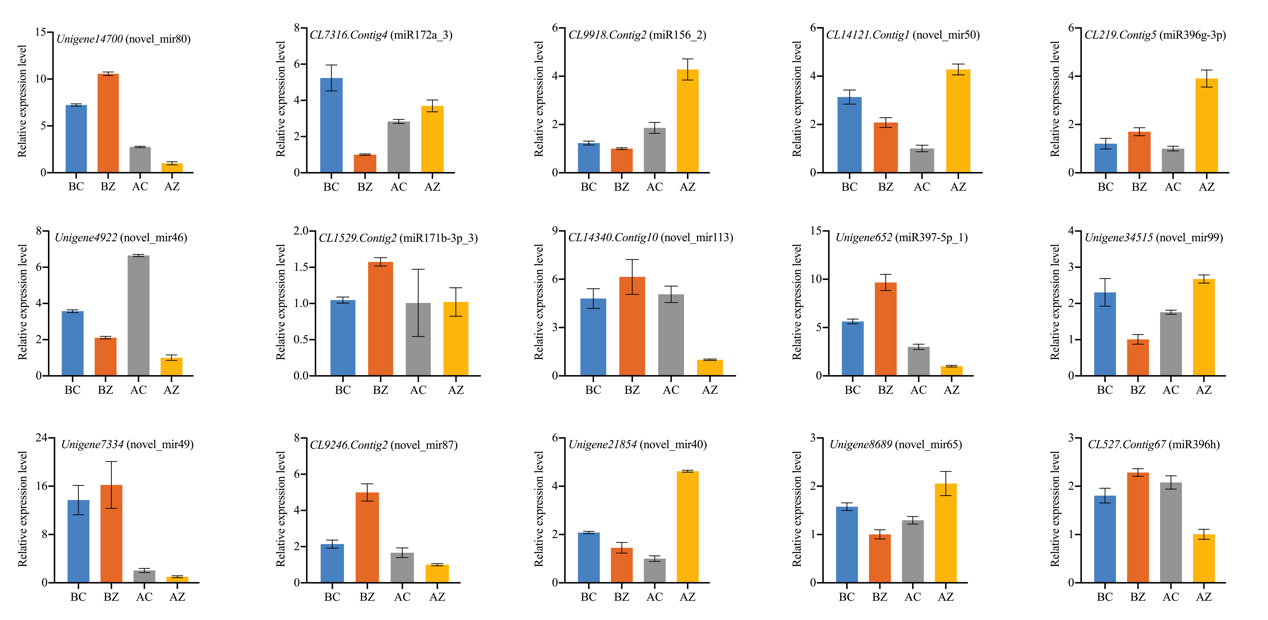
**Figure S7. qRT-PCR analysis of miRNAs target genes in *M. savatieri*.** The error bars of qRT-PCR data indicate the standard deviations of the three replicate determinations.

**Table S2 Specific primers of differentially expressed genes for qRT-PCR validation.**

| Number | Gene | Primer | Sequence (5'-3') |
| --- | --- | --- | --- |
| 1 | Unigene11723 | F | ATCAACGCCGCCACATCACA |
|  |  | R | CTGGTTAGCGGCCTGAGACG |
| 2 | Unigene28565l | F | TGCTTGCTGTGCGACACTGA |
|  |  | R | TCGGATTTGCCCTTCGCCTC |
| 3 | Unigene25653 | F | ACGCGAGTCATGCTCAGCTC |
|  |  | R | TCACGACCTTCACCCCGTCT |
| 4 | Unigene35991 | F | GCGGCACCTTCGTCTCTCTC |
|  |  | R | TCACCGTTGCTCTCATCGCC |
| 5 | Unigene32595 | F | AACTGGGACAAGGGCAAGGC |
|  |  | R | ACGTCGCAAGAACGCCATGA |
| 6 | Unigene9451 | F | GCTGGCTCTGTCCGTTTGGT |
|  |  | R | TCGGGACTTGCTCATTCGCC |
| 7 | Unigene21852 | F | ATGCCAGCACTCACCGGTTT |
|  |  | R | AACTCCTCAGCGCCTTGCTC |
| 8 | Unigene29438 | F | GGATCCGGCAAATGGTCCGT |
|  |  | R | GTGGCAGCATCTCAGCGGAT |
| 9 | Unigene60184 | F | CGTCACAGCAGGCAGAGGAG |
|  |  | R | CGAGCTTGCCCAGTGCGATA |
| 10 | Unigene36901 | F | CATGCCAGCACTCACCGGAT |
|  |  | R | TCAGCGCCTTGCTCCAAACA |
| 11 | Unigene470 | F | AACTGTTGTCCCTGGCGGTG |
|  |  | R | TCCTCCCTGGCCTCCGAAAA |
| 12 | Unigene5067 | F | TGCCCATTGTTCTCGTGGGG |
|  |  | R | CCGCGTCGAACACTGCCTTA |
| 13 | Unigene10023 | F | GGCACATGGGGCTAAGCGAT |
|  |  | R | GGCAGCCGGATCAGACCTTC |
| 14 | Unigene36013 | F | GGCTGGTTTGGACTTGGGCT |
|  |  | R | ACCTCCAATATGTGCGCCCG |
| 15 | Unigene25917 | F | GCAAGAGCTCGTGAGCTGGA |
|  |  | R | TCCCACCATTTCGACGTGCC |
| Reference | Actin | F | AGCCCGAGCCTCAATCCATC |
|  |  | R | TGGAGGGCTGCCCATTAAGC |
| Reference | GAPDH | F | TCCCTTGGGGTGACTACGGG |
|  |  | R | GACCTTCTTGGCACCACCCC |

**Table S3 Specific primers of differentially expressed miRNAs for qRT-PCR validation.**

| Number | miRNA | Primer | Sequence (5'-3') |
| --- | --- | --- | --- |
| 1 | novel_mir99 | F | CGCTCGATCGTCTAACGTGTTAGATG |
| 2 | miR397-5p_1 | F | CGCATTGAGTGCAGCGTTGATGA |
| 3 | novel_mir113 | F | GTTCGATTCTCGTATGGTGCAGTTTCCTCA |
| 4 | novel_mir50 | F | CGATTGAATCTCCCGTTGTTCCTCAGTA |
| 5 | miR171b-3p_3 | F | CTTGAGCCGTGCCAATATCACG |
| 6 | novel_mir46 | F | TATGGAGTCCGGAGATGCTGG |
| 7 | miR172a_3 | F | CGCCAGAATCTTGATGATGCTGCAT |
| 8 | miR396h | F | CGCCTCCACAGCTTTCTTGAACT |
| 9 | novel_mir80 | F | CGCAAGGCATGTGTAGGATAGGTG |
| 10 | miR156_2 | F | CGCGCTTGACAGAAGATAGAGAGC |
| 11 | miR396g-3p | F | CGCGTTCAAGAAAGCTGTGGAAGA |
| 12 | novel_mir40 | F | CGCGCCGTTGAGCTTGTTTAATTGAT |
| 13 | novel_mir87 | F | CGTGTGGATTCGAGTCATGTGCT |
| 14 | novel_mir65 | F | CGCGCGCGCGTAAATTGATTTTTTTTT |
| 15 | novel_mir49 | F | CGCTGAGTCACCATAATTTACCTCCTC |
| Reference | U6snRNA | F | TGGAACGCTTCACGAATTTGCG |
|  |  | R | GGAACGATACAGAGAAGATTAGC |

**Table S4 Specific primers of target genes of differentially expressed miRNAs for qRT-PCR validation.**

| Number | Target gene | Primer | Sequence (5'-3') | miRNA |
| --- | --- | --- | --- | --- |
| 1 | Unigene34515 | F | GGTCCAACAAGCTTCGGCCT | novel_mir99 |
|  |  | R | TGTGGGGGTGATAAGGGAGTCA |  |
| 2 | Unigene652 | F | TGACAACACCACGACCACCG | miR397-5p_1 |
|  |  | R | AAGAACTTGTGGGCCGTGGG |  |
| 3 | CL14340.Contig10 | F | GGAGGAATCCTGGGGTGGGT | novel_mir113 |
|  |  | R | TGTCGCCAAATGGACTGCCA |  |
| 4 | CL14121.Contig1 | F | ACCTTCTGCGGCCAATGCTT | novel_mir50 |
|  |  | R | GGGTGGGTCCCTCCATAGCA |  |
| 5 | CL1529.Contig2 | F | GGAAAGGAACTGCCTCCGGG | miR171b-3p_3 |
|  |  | R | ATCGCGTTTATGGCGACGGT |  |
| 6 | Unigene4922 | F | CCGAACGCCCTCGTCTCTTC | novel_mir46 |
|  |  | R | TCAACGACTCGCCCCTCTCA |  |
| 7 | CL7316.Contig4 | F | GGATTGGTCGAGGGGATGCG | miR172a_3 |
|  |  | R | AAACTCCCAGCAAACCGCGA |  |
| 8 | CL527.Contig67 | F | GGGAGTTGGATGAGCTGAGGC | miR396h |
|  |  | R | CCCGTGTCTTTCCAGTGCGA |  |
| 9 | Unigene14700 | F | CCCTAACCGCCAGCTACTGC | novel_mir80 |
|  |  | R | CGCAGCTCGCTCATCTGGAA |  |
| 10 | CL9918.Contig2 | F | GTGCAGGTGGATCGGGACAG | miR156_2 |
|  |  | R | TTGGCAGAAGCGTTGCCTCA |  |
| 11 | CL219.Contig5 | F | CGCTAGCGCTACAGCAACCT | miR396g-3p |
|  |  | R | TGAGAGCGCTGGCGAAGATG |  |
| 12 | Unigene21854 | F | CATGCCAGCACTCACCGGAT | novel_mir40 |
|  |  | R | TCAGCGCCTTGCTCCAAACA |  |
| 13 | CL9246.Contig2 | F | AAACCACGGTGGGGTTCGAC | novel_mir87 |
|  |  | R | CCTTACTGGCCATGCCCTCG |  |
| 14 | Unigene8689 | F | TCTGCATCGCAAAGCACTGA | novel_mir65 |
|  |  | R | GGGTCGAGGAAGAGTGCAGC |  |
| 15 | Unigene7334 | F | AGCCGAGCTCGAACAACAGG | novel_mir49 |
|  |  | R | ACGCGCAGATACCAATGGGG |  |
| Reference | Actin | F | AGCCCGAGCCTCAATCCATC | / |
|  |  | R | TGGAGGGCTGCCCATTAAGC |  |
| Reference | GAPDH | F | TCCCTTGGGGTGACTACGGG | / |
|  |  | R | GACCTTCTTGGCACCACCCC |  |
